# Single‐base methylome analysis reveals dynamic epigenomic differences associated with water deficit in apple

**DOI:** 10.1111/pbi.12820

**Published:** 2017-09-22

**Authors:** Jidi Xu, Shasha Zhou, Xiaoqing Gong, Yi Song, Steve van Nocker, Fengwang Ma, Qingmei Guan

**Affiliations:** ^1^ State Key Laboratory of Crop Stress Biology for Arid Areas College of Horticulture Northwest A&F University Yangling Shaanxi China; ^2^ Department of Horticulture Michigan State University East Lansing MI USA

**Keywords:** apple, methylomes, epigenetics, gene expression, water deficit, transcriptome

## Abstract

Cytosine methylation is an essential feature of epigenetic regulation and is involved in various biological processes. Although cytosine methylation has been analysed at the genomic scale for several plant species, there is a general lack of understanding of the dynamics of global and genic DNA methylation in plants growing in environments challenged with biotic and abiotic stresses. In this study, we mapped cytosine methylation at single‐base resolution in the genome of commercial apple (*Malus* x *domestica*), and analysed changes in methylation patterns associated with water deficit in representative drought‐sensitive and drought‐tolerant cultivars. We found that the apple genome exhibits ~54%, ~38% and ~8.5% methylation at CG, CHG and CHH sequence contexts, respectively. We additionally documented changes in gene expression associated with water deficit in an attempt to link methylation and gene expression changes. Global methylation and transcription analysis revealed that promoter‐unmethylated genes showed higher expression levels than promoter‐methylated genes. Gene body methylation appears to be positively correlated with gene expression. Water deficit stress was associated with changes in methylation at a multitude of genes, including those encoding transcription factors (TFs) and transposable elements (TEs). These results present a methylome map of the apple genome and reveal widespread DNA methylation alterations in response to water deficit stress. These data will be helpful for understanding potential linkages between DNA methylation and gene expression in plants growing in natural environments and challenged with abiotic and biotic stresses.

## Introduction

Cytosine methylation is a common feature of eukaryotic genomes and is associated with various genetic processes including regulation of transcription, silencing of transposable elements (TEs), genome integrity and imprinting (Cedar and Bergman, 2012). In plants, cytosine methylation is found in three sequence contexts: CG, CHG and CHH (where H = A, T or C; Law and Jacobsen, 2010). In each of these contexts, distinct mechanisms are involved in establishing, maintaining and removing the methylation mark. For symmetrical methylation, CG methylation is catalysed by Methyltransferase1 (MET1). CHG methylation is established predominately by the activity of the plant‐specific enzyme Chromomethylase 3 (CMT3; Lindroth *et al*., 2001). Additionally, a class of histone methyltransferases (KRYPTONITE (KYP/SUVH4), SUVH5 and SUVH6) are required for CMT3 DNA methylation activity (Jackson *et al*., 2002; Lindroth *et al*., 2004). For methylation at nonsymmetrical (CHH) sites, two proteins designated Domains Rearranged Methyltransferases (DRM)1 and DRM2 are the enzymatic activities responsible for asymmetric CHH methylation. In this process, methylation is guided by small (typically 24 nucleotide) RNAs called small interfering RNAs (siRNAs), which are processed from precursor RNAs by Dicer‐like 3 (DCL3; Matzke and Mosher, 2014). Maintenance of cytosine methylation after replication for all three sequence contexts also depends on the chromatin remodelling protein Decrease in DNA Methylation 1 (DDM1; Jeddeloh *et al*., 1999). Although this maintenance is typically very robust, cytosine methylation can also be erased by a class of DNA glycosylases, including DEMETER (DME), REPRESSOR OF SILENCING 1 (ROS1), DEMETER‐LIKE 2 and DML3 (Choi *et al*., 2002; Gong *et al*., 2002; Penterman *et al*., 2007).

Environmental abiotic stresses such as drought, heat, cold and high salinity affect plant growth, productivity and distribution. Survival in response to abiotic stress involves various physiological, biochemical and genetic responses (Hirayama and Shinozaki, 2010). Recently, it is becoming clear that epigenetic factors contribute to transcriptional and post‐transcriptional regulations of gene expression important for stress responses (Mirouze and Paszkowski, 2011). A number of recent studies have revealed that DNA methylation in modulation of gene expression involves in both biotic (Dowen *et al*., 2012) and abiotic stresses (Sahu *et al*., 2013). In rice, drought stress‐induced genome‐wide DNA methylation alterations and 70% of drought‐induced methylation changing sites were reversed to their original status after water recovery (Wang *et al*., 2011). Moreover, induction of the rice *OsMYB91* gene in response to salt stress was associated with rapid loss of promoter cytosine methylation, suggesting that dynamic methylation may have a role in modulating the expression of this gene (Zhu *et al*., 2015). In addition, single‐base resolution methylome analysis of drought‐stressed poplar (*Populus trichocarpa*) revealed widespread changes in DNA methylation (Liang *et al*., 2014). Another study of diverse poplar genotypes found that the amplitude of drought‐related transcriptional changes was reflected by the extent of genomic changes in DNA methylation, suggesting the importance of epigenetic mechanisms in adaption of long‐lived trees to local environment (Brautigam *et al*., 2013). In addition, environmental stresses on plants also induced epigenetic variations in transposable element (TE) locus, suggesting the involvements of TEs in stress response with epigenetic changes (Sahu *et al*., 2013). For example, transcriptional silencing of the maize ZmMET1 gene in roots in response to cold led to demethylation of the Ac/Ds transposon region (Steward *et al*., 2000). In snapdragon (*Antirrhinum majus*), the *Tam3* transposon showed altered methylation at CHH sites under low‐temperature stress (Hashida *et al*., 2006). Similarly, the *Mutator* element *MuDR* in maize was demethylated with the increasing expression level of mudrA transposase gene and mudrB helper gene in response to low‐energy nitrogen ion (N^+^) implantation (Qian *et al*., 2010). These studies revealed a direct relationship between environmental stresses, changes in DNA methylation and TE mobilization.

Apple (*Malus* × *domestica* Borkh.) is one of the most important fruit crops in temperate regions. As a long‐lived and woody perennial tree species, apple is affected by numerous environmental stresses, leading to the economic losses. Therefore, understanding the mechanisms of abiotic stress response in apple is important for guiding management of genetic resources and plant breeding. Studies of epigenomic variation in plants have been largely confined to a small number of model organisms, such as *Arabidopsis* and rice. However, the rapid development of high‐throughput sequencing technologies has now enabled the mapping of cytosine methylation at the genomic scale in species such as apple with large and complex genomes (Lister *et al*., 2008; Niederhuth *et al*., 2016; Zhong, 2016). To date, there have been no studies of the influences of environmental factors such as water deficit stress on the methylome in apple.

In this study, we generated single‐base resolution and high‐coverage genome‐wide maps of cytosine methylation in two apple cultivars with contrasting drought stress tolerance. The study was designed to gain insights into three questions: (i) the genomic landscape of the apple methylome, (ii) changes in the apple methylome associated with water deficit and (iii) evaluation of the relationship between methylome changes and water deficit‐associated gene expression changes.

## Results

### Genes encoding potential DNA methyltransferase and demethylase are induced by drought stress

**Figure 1:**
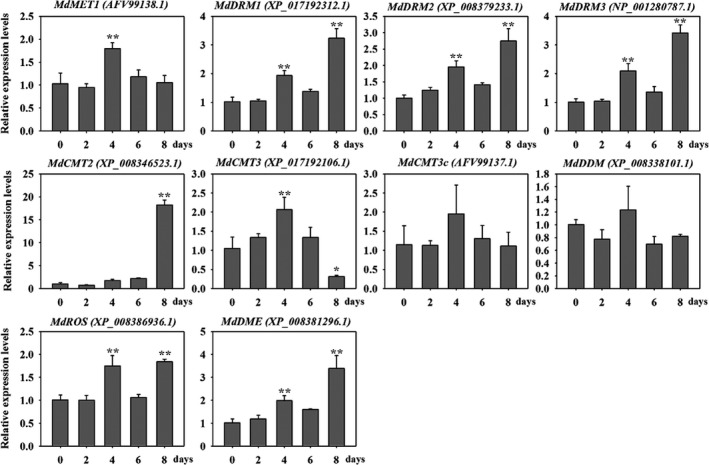
Relative expression analysis of genes encoding homologs of DNA methyltransferase and demethylases under drought stress for 2, 4, 6 or 8 days using real‐time PCR. Transcripts of *Malus* elongation factor 1 alpha gene (EF‐1α, DQ341381) were used as an endogenous control to normalize expression in different samples. Bars represented means ± SD from three biological replicates. Each biological replicate was performed with independent RNA extractions and two technical replicates. Significant differences calculated from the three biological replicates were indicated by *(*P* value < 0.05) and **(*P* value < 0.01).

Cytosine methyltransferases and demethylases play crucial roles in maintenance of genomic methylation and participate in a variety of biological processes. To identify potential methyltransferases and demethylases in apple, we globally inspected the methylation‐related genes encoding DNA methyltransferases and demethylases at the genome scale (Table S1). For MET1, we found one MET1 in apple genome MdMET1 (XP_008361333.1), which showed 92% identities with previously submitted protein AFV99138.1 (GenBank). Except CMT1, apple genome contained two CMT2 homologs and three CMT3 homologs. For DRM family, we found one DRM1 counterpart and four putative DRM2 proteins in apple genome. More details are shown in Table S1. The expression patterns of these genes were further analysed in ‘Golden Delicious’ leaves in response to drought stress for 2, 4, 6 or 8 days (Figure 1). *MdDRM* members showed the similar expression patterns and presented highest expression level at 8 days under drought stress. The putative *MdCMT2* showed highest expression after 8 days. However, the expression level of *MdCMT3* increased during the first 4 days and then decreased at 8 days. Notably, the mRNA level of demethylase gene *MdDME* showed generally increasing expression trend (Figure 1). These results show that drought is associated with changes in transcriptional levels of genes with potential roles in DNA methylation.

### Methylation landscapes in apple

**Figure 2:**
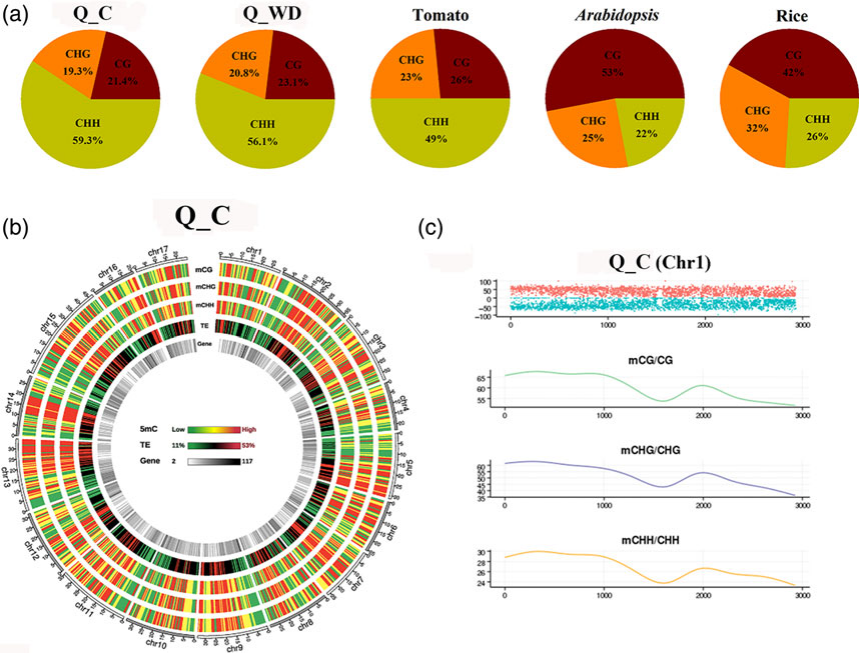
The apple epigenome. (a) Relative proportions of mCs in three sequence contexts (CG, CHG and CHH) in apple, tomato, *Arabidopsis* and rice. (b) Circos plots of chromosomes in apple genome. Track order: density plot of 5mC in CG, CHG and CHH contexts; density of transposable elements (TEs); gene density of each chromosome. (c) Distributions of 5‐methylcytosine density on chromosome 1. Q_C, ‘Qinguan’ control; Q_WD, ‘Qinguan’ water deficit.

The observed changes in expression of methylation‐controlling genes suggested that genomic DNA methylation patterns may be altered in response to drought. To investigate the overall methylation patterns of apple responding to water deficit, two apple cultivars, drought‐tolerant ‘Qinguan’ (‘QG’) and drought‐sensitive ‘Honeycrisp’ (‘HC’), were subjected to water deficit treatment by plants growing in field environments (Figure S1a). Then, the corresponding methylomes were decoded and analysed. Up to ~300 million sequencing reads were generated for each replicate, yielding ~37–40 Gb of data representing >20× of the NCBI reference genome (49× of the estimated genome size 742.3 Mb; Table 1). The Q_C (‘Qinguan’ control) genome presented ~18% (mCs), 54% (mCG), 38% (mCHG) and 8.5% (mCHH) on the total sequenced C sites, CG, CHG and CHH sites, respectively, which reflected the percentage of methylation levels in the genome. Accordingly, H_C (‘Honeycrisp’ control) presented ~17%, 50%, 34% and 8.3% in C, CG, CHG and CHH contexts (Table S2, Figure S3). Moreover, we also analysed the proportion of mCG, mCHG and mCHH on the total mC sites, which reflected the distributions of mCs in three sequence contexts. As shown in Figure 2a, methylcytosine was most often found at CHH sites (59.3%), with less frequent occurrences at CG and CHG sequences (21.4% and 19.3%, respectively). Water deficit was associated with a slight increase in CG and CHG methylation proportions, and decrease in CHH methylation in both ‘QG’ and ‘HC’ (Figures 2a and S2b). From the global perspective, we observed a high degree of methylation within TE‐rich regions in apple genome. In contrast, gene‐rich regions with low density of TEs were characterized by reduced methylation levels (Figure 2b). The density of mC for chromosome 1 is shown in Figure 2c. Distribution of mCs identified on the sense and antisense strands of all 17 apple chromosomes for each sequence context is presented in Figure S4. The methylation landscapes were almost identical between ‘QG’ and ‘HC’ genomes, suggesting that the methylation landscape is similar between ‘QG’ and ‘HC’ genome from the genome‐wide perspective. Therefore, we used ‘QG’ methylome data to exhibit the apple methylation landscapes and ‘HC’ methylome is shown in Figure S2 in detail.

**Table 1:** Data description of BS‐Seq reads for the four apple samples with two replicates

Samples	Raw reads/Data production (Gb)	Clean reads/Data production (Gb)	BS conversion rate	Total reads	Mapped reads	Mapping rate (%)	Sequencing depth
Q_C1	305 487 614/38.19	302 394 290/37.8	99.89%	151 197 145	99 164 028	65.59	10.9
Q_C2	308 269 296/38.53	305 348 696/38.17	99.88%	152 674 348	97 670 677	63.97	10.6
Q_WD1	293 923 682/36.74	291 051 260/36.38	99.88%	145 525 630	94 562 992	64.98	10.2
Q_WD2	297 303 286/37.16	294 461 824/36.81	99.87%	147 230 912	96 185 179	65.33	10.4
H_C1	313 776 256/39.22	310 753 798/38.84	99.88%	155 376 899	99 049 711	63.75	10.6
H_C2	297 583 798/37.2	294 123 798/36.77	99.90%	147 061 899	94 917 926	64.54	10.2
H_WD1	318 890 518/39.86	304 688 444/38.09	99.87%	152 344 222	96 899 509	63.61	11.1
H_WD2	301 204 726/37.65	290 502 288/36.31	99.87%	145 251 144	92 684 611	63.81	10.6

Q_C1/2 represents ‘Qinguan’ control replicate 1/2 and Q_WD1/2 represents ‘Qinguan’ water deficit treatment replicate 1/2. H_C1/2 represents ‘Honeycrisp’ control replicate 1/2 and H_WD1/2 represents ‘Honeycrisp’ water deficit treatment replicate 1/2.

### DNA methylation patterns in different apple genomic regions

**Figure 3:**
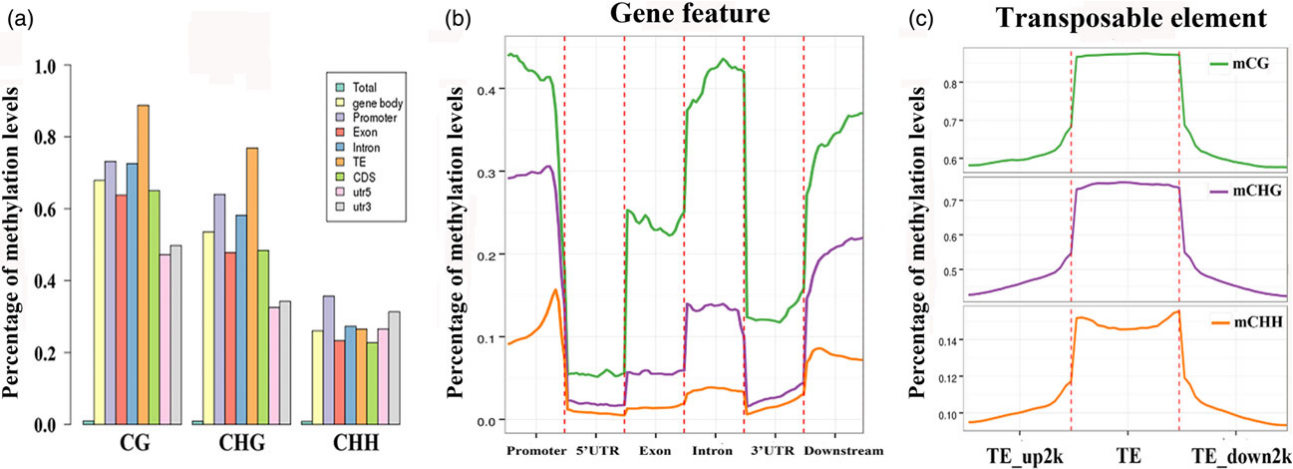
DNA methylation patterns in different genomic regions. (a) Percentage of methylation levels of promoter, transposable element (TE), coding genes with 5′UTR, exon, intron, CDS, 3′UTR regions. (b) Distributions of DNA methylation levels among gene features, including promoter, 5′UTR, exon, intron, 3′UTR and downstream 2 kb. (c) Percentage of methylation levels among TE regions and their 2‐kb upstream and downstream regions.

The DNA methylation statuses varied among the different genomic regions, such as euchromatin and heterochromatin, repetitive sequences and coding sequences. To explore the DNA methylation patterns in different apple genomic regions, we analysed the methylation profiles within genes, including coding sequences and introns. The CG context presented the highest methylation level and CHH was the lowest among three contexts in each gene region (Figure 3a). For CG context, CG methylation within 5′UTR and 3′UTR regions was much lower than the CDS regions, and methylation levels in exon and CDS were much lower than the promoter and intron regions. TE showed highest methylation level among these regions. Methylation level in CHG context presented similar patterns with CG context. However, for CHH context, CHH methylation showed the highest level in promoter region and lowest in CDS and exon regions (Figure 3a).

We also evaluated methylation levels within transcribed regions and ~2 kb upstream and downstream regions. As shown in Figure 3b, CG and CHG showed methylation changes among the gene features. CG and CHG methylation was highest in promoters regions, lowest in the 5′UTR, increased within the transcribed regions but declined among the 3′UTR regions, then rises back in 2‐kb downstream regions (Figure 3b). As for TE region, consistent with studies in *Arabidopsis* and rice, each of CG, CHG and CHH sequence contexts shows high methylation enrichments in TE regions with comparative low methylation status among 2‐kb upstream and downstream regions (Figure 3c). Hypermethylation of TE regions is probably responsible for suppression of active transposons.

### Correlation between DNA methylation status and gene expression levels

**Figure 4:**
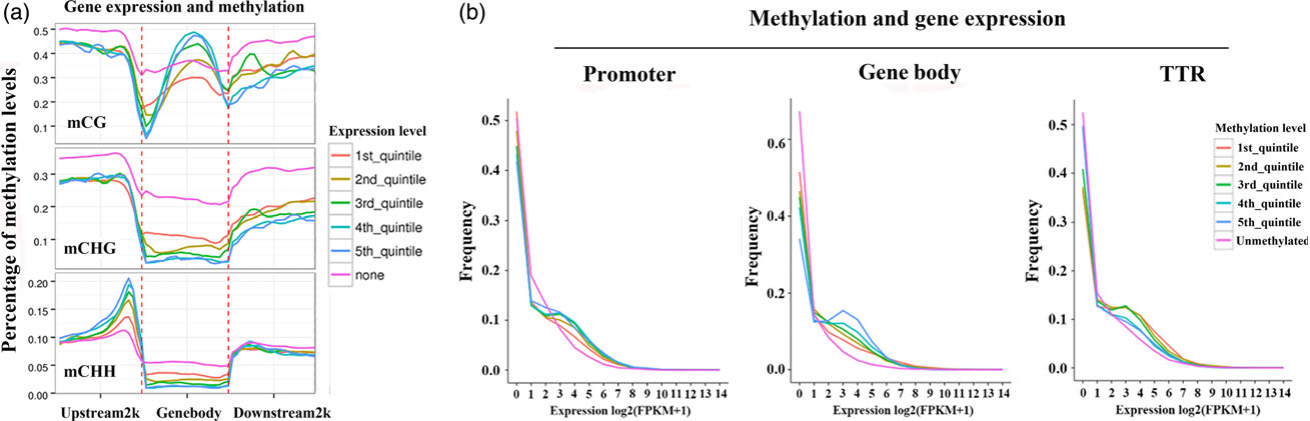
Relationship between DNA methylation and gene expression. (a) Distributions of methylation levels within gene bodies partitioned by different expression levels: 1st_quintile is the lowest and 5th_quintile is the highest; genes with FPKM value <0.1 were considered nonexpressed (none). (b) Expression profiles of methylated genes compared with unmethylated genes. Methylated genes were further divided into quintiles based on promoter, gene body and transcriptional termination region (TTR) methylation levels: 1st_quintile is the lowest and 5th_quintile is the highest.

DNA methylation regulates genes in a wide variety of biological processes. To reveal potential regulatory roles of promoter and gene body methylation for gene expression, transcriptome profiles were generated with ‘QG’ and ‘HC’ under water deficit, the same materials as methylome analysis. Genes were firstly divided into nonexpressed (none) genes (FPKM value < 0.1) and expressed genes. The latter were further divided into five groups based on expression level, with the first quintile having the lowest expression level and the fifth quintile having the highest. As expected, nonexpressed genes showed relatively high methylation levels in all three sequence contexts (Figure 4a). However, genes with the highest expression levels showed high CG methylation levels throughout much of the gene body but relatively low methylation near the transcription start and end sites. In contrast, CHG methylation levels were inversely correlated with expression throughout most regions of the gene (Figure 4a). Interestingly, CHH methylation levels in the promoter region were positively correlated with expression. In contrast, gene body CHH methylation was negatively correlated with expression. However, there were no distinct differences in the downstream 2‐kb region (Figure 4a).

To further assess the relationship between gene methylation and gene expression, genes were classified as methylated or unmethylated based on methylation level. Methylated genes were rank‐ordered based on the degree of promoter or gene body methylation and divided into quintiles (Figure 4b). The first quintile is the 20% of genes with the lowest methylation level, and the fifth group is the highest. As shown in Figure 4b, genes lacking promoter methylation showed higher expression levels than methylated genes, but no obvious difference was observed among the five methylation‐level quintiles. Consistent with these results, gene body methylation appears to be positively correlated with gene expression as higher levels of gene body methylation show higher expression levels. In addition, genes lacking methylation within the gene body show relatively lower expression levels compared with the other methylation quintiles (Figure 4b). Previous studies revealed that methylation within TTR (transcriptional termination region) could repress gene expression (Li *et al*., 2012). Therefore, we also analysed the association of different TTR methylation status with their expression levels. As shown in Figure 4b, the five methylation‐level quintiles showed negative correlation with the expression levels, consistent with the previous results. Unexpected, the unmethylated TTR showed the lowest expression levels compared with the five methylation‐level groups.

### Methylation profiles of functional genes and transposable elements in apple

In order to gain an insight into the functional distinctions between methylated and unmethylated genes, we used GO (Gene Ontology) analysis to categorize the methylated and unmethylated genes (Figure S5). Methylated genes were enriched in binding activity terms, including cyclic compound binding and nucleic acid binding. As for biological processes, methylated genes are enriched for cellular metabolic and biosynthetic processes. In contrast, unmethylated genes are enriched in lipid biological process, DNA‐related activities, and kinases (Figure S5).

**Figure 5:**
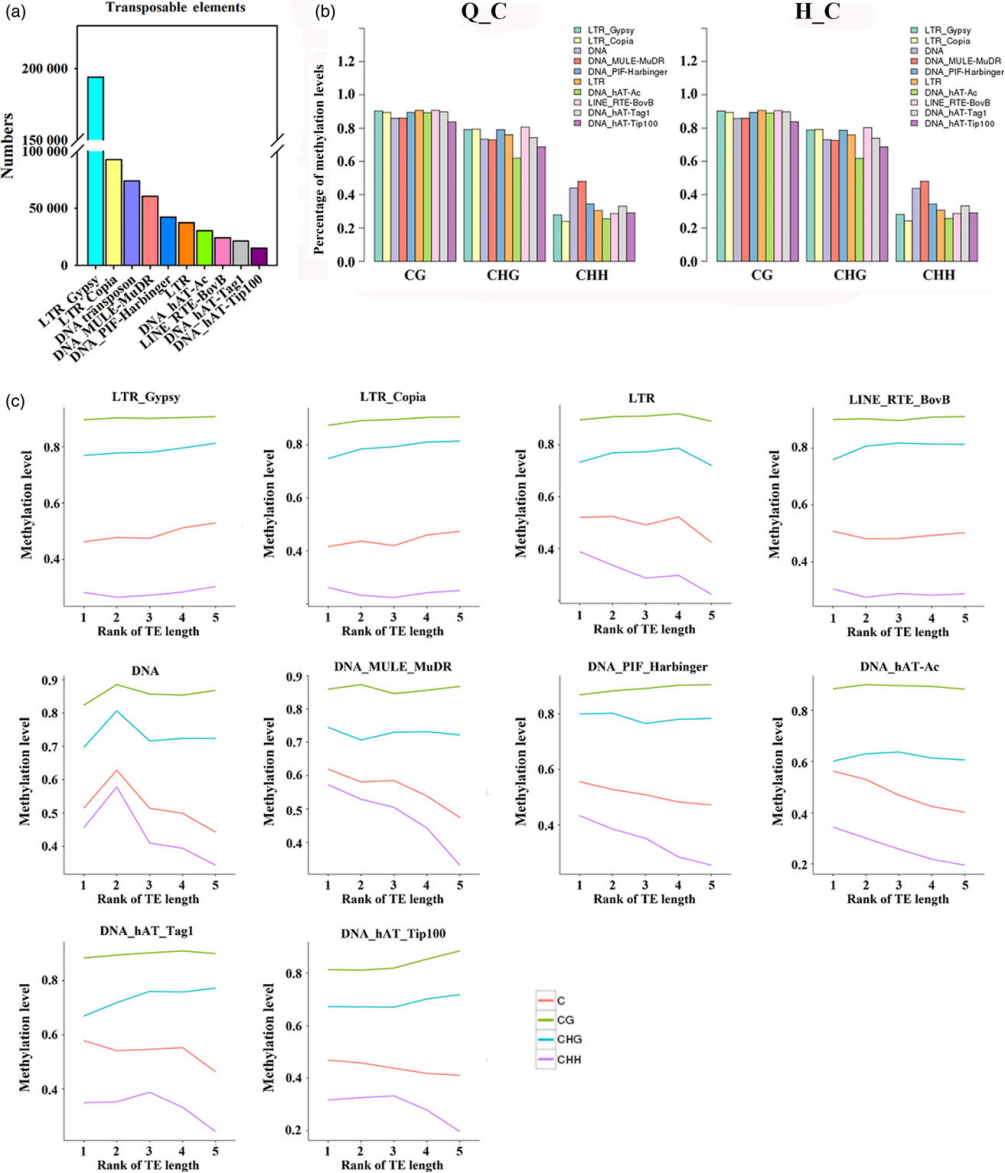
Methylation patterns of transposable elements in the apple genome. (a) Numbers of different types of TEs in apple genome. (b) Percentage of methylation levels of different types of TEs in ‘Qinguan’ and ‘Honeycrisp’. (c) Methylation patterns of different types of TEs with different length. TEs divided into quintiles based on their length: 1st_quintile is the shortest and 5th_quintile is the longest length of TEs. Q_C, ‘Qinguan’ control; H_C, ‘Honeycrisp’ control.

Transposable elements (TEs) can affect genome size, create mutations with insertion and excision, and affect gene expression. To characterize the methylation profiles of transposable elements in apple, we first identified members of the various classes of TEs in the apple genome (Figure 5a). Among these, Gypsy and Copia LTR‐retrotransposons were the most numerous (194 115 LTR‐Gypsy and 92 618 LTR‐Copia). Then, the methylation level of these TEs was analysed (Figure 5b). TEs presented high methylation levels in CG and CHG contexts and different types of TEs show similar CG and CHG methylation levels, with only minor differences in CHH methylation (Figure 5b). Previous studies revealed that methylation levels are also associated with the TE length (Xing *et al*., 2015). To reveal the relationship between TE length and methylation status in apple, TEs were divided into five quintiles based on the TE length: the first quintile is the 20% of TEs with the shortest length and the fifth group is the longest. As shown in Figure 5c, the most numerous TEs, Gypsy and Copia, showed marginally increasing CHG and CHH methylation with increasing length. In contrast, other LTR transposons showed a striking decrease in CHH methylation with length. DNA TEs showed the highest methylation level in all three sequence contexts in moderate length TEs (2nd_quintile). Methylation levels of hAT_Tag1 and hAT‐Tip100 increased with TE length at CG and CHG contexts, but decreased at CHH contexts (Figure 5c).

### Widespread dynamic DNA methylation in response to water deficit stress

**Figure 6:**
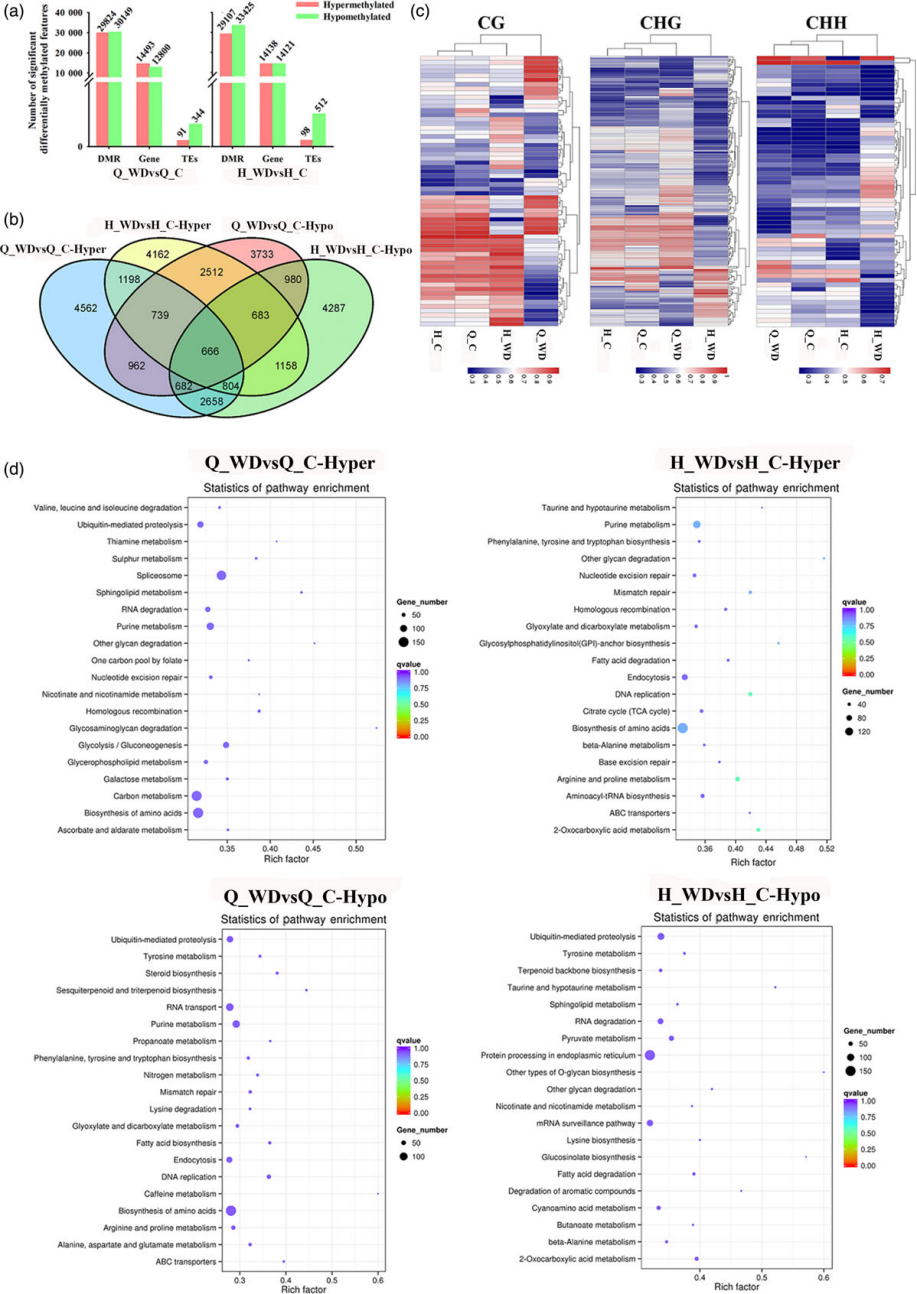
Differential methylome analysis under water deficit stress. (a) Numbers of differentially methylated regions (DMR), genes (DMGs) and TEs. (b) Venn diagram of hyper/hypomethylated genes among ‘Qinguan’ and ‘Honeycrisp’ under water deficit stress. (c) Heat maps of methylation levels within CG, CHG and CHH DMRs, respectively; (d) KEGG pathway enrichment of hypermethylated and hypomethylated genes in two cultivars under water deficit. The size of the circle represents gene numbers, and the colour represents the *q*‐value. Q_WDvsQ_C, ‘Qinguan’ water deficit versus ‘Qinguan’ control; H_WDvsH_C, ‘Honeycrisp’ water deficit versus ‘Honeycrisp’ control.

**Figure 7:**
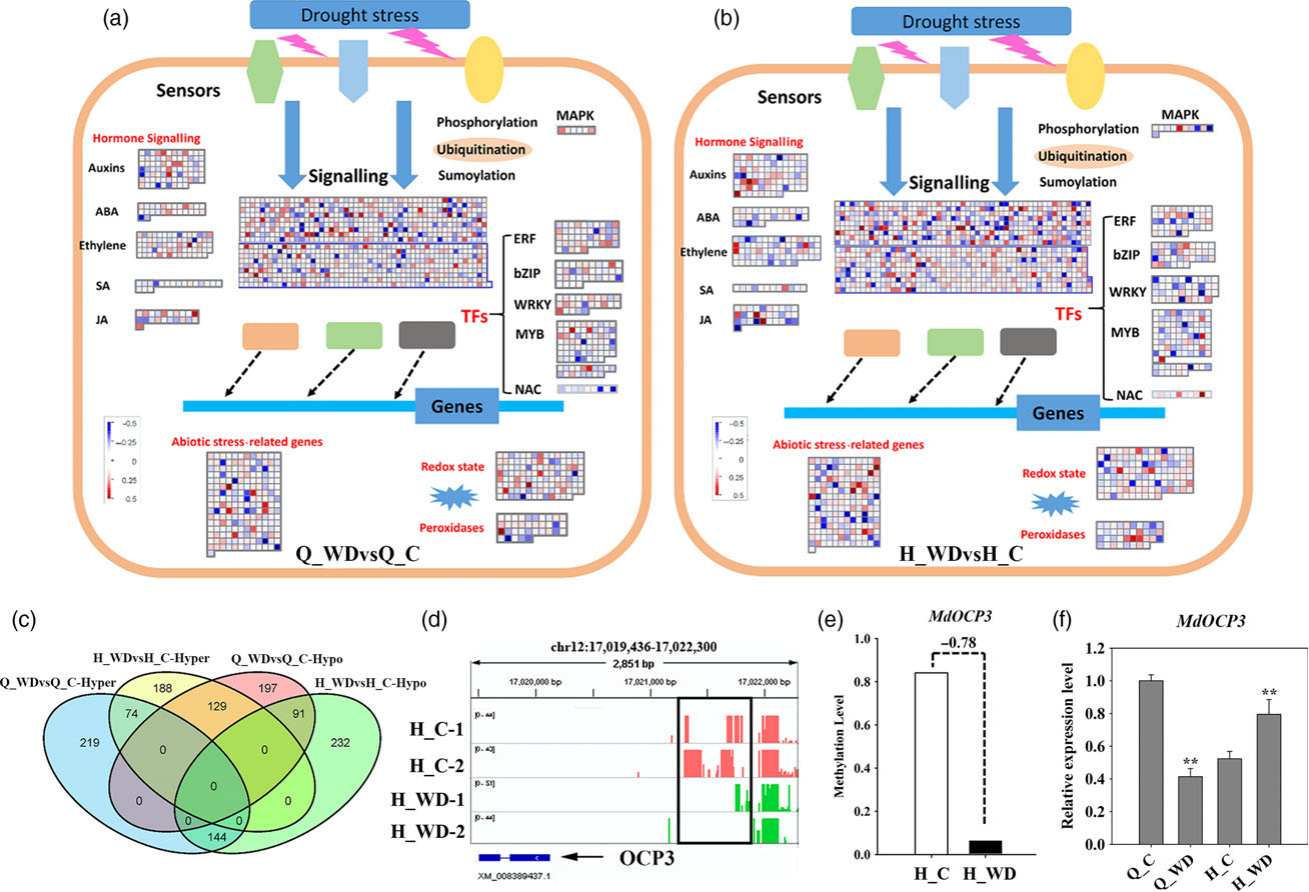
Assignment of differentially methylated genes among ‘Qinguan’ (a) and ‘Honeycrisp’ (b) under water deficit in Mapman bins. The red and blue squares indicated the hyper‐ and hypomethylated genes. (c) Venn map of differentially methylated transcriptional factors (TFs); (d) IGV software depicts the demethylation of *OCP3* promoter region induced by water deficit stress in ‘Honeycrisp’; (e) the methylation difference of *MdOCP3* in ‘Honeycrisp’ under water deficit; (f) expression analysis of *MdOCP3* in ‘Qinguan’ and ‘Honeycrisp’ under water deficit. Q_WDvsQ_C, ‘Qinguan’ water deficit versus ‘Qinguan’ control; H_WDvsH_C, ‘Honeycrisp’ water deficit versus ‘Honeycrisp’ control. Significant differences calculated from the three replicates were indicated by ** (*P* value < 0.01).

To investigate the potential influence of water deficit stress on methylation, we analysed the DMRs (differentially methylated regions) between Q_WDvsQ_C and H_WDvsH_C (‘QG'_water deficit versus control and ‘HC'_water deficit versus control). The distribution of DMR length on each chromosome and methylation levels of DMRs are shown in Figure S6. In total, 29 824 hypermethylated and 30 149 hypomethylated DMRs were identified in Q_WDvsQ_C, and these overlapped with 14 493 hypermethylated and 12 800 hypomethylated genes, respectively. Similarly, 29 107 hyper‐DMRs containing 14 138 hypermethylated genes and 33 425 hypo‐DMRs containing 14 121 hypomethylated genes were identified in H_WDvsH_C (Figure 6a). ‘QG’ and ‘HC’ shared 3407 hypermethylated genes and 3011 hypomethylated genes under water deficit stress (Figure 6b). The heat maps also presented a widespread methylome change under water deficit stress (Figure 6c). In order to investigate the biological functions of DMR genes, a KEGG (Kyoto Encyclopedia of Genes and Genomes) pathway enrichment analysis was performed. As shown in Figure 6d, hypermethylated genes in Q_WDvsQ_C are mainly assigned in pathways related to spliceosome, carbon metabolism, purine metabolism, biosynthesis of amino acids, etc. The last two pathways are also enriched in H_WDvsH_C. Among hypomethylated genes in Q_WDvsQ_C, pathways of RNA transport and biosynthesis of amino acids were enriched. However, hypomethylated genes in H_WDvsH_C show abundant enrichment on the pathway of protein processing in the endoplasmic reticulum pathway. Notably, the two cultivars share the ‘ubiquitin‐mediated proteolysis’ enrichment, suggesting that water deficit stress alters the methylation status of genes involved in ubiquitin‐mediated proteolysis and may lead to specific protein degradation (Figure 6d). As shown in Figure S7, genes encoding E1 activation proteins were demethylated. The methylation levels of abundant F‐box (SCF) and E3 ligase RING genes were disrupted. Figure 7a and b shows the detail assignations of differentially methylated genes (DMGs) in pathways by MapMan. Abundant DMGs were involved in ‘signalling’ pathway. Genes involved in hormone signalling pathways, including auxins, ABA (abscisic acid), ethylene, SA (salicylic acid), JA (jasmonic acid), were also differentially methylated. Moreover, water deficit‐related transcription factors including *AP2/EREBP*,* bZIP*,* WRKY*,* MYB* and *NAC* members showed methylation variations. Additionally, the methylation levels of genes involved in abiotic stress, redox and peroxidase genes were also disrupted (Figure 7a and b).

To characterize the methylation alterations of transcription factors (TFs) under water deficit stress, we firstly predicted 3698 TFs from apple genome using PlantTFDB. About 850 TFs showed changes in methylation in Q_WDvsQ_C. These included 437 hypermethylated genes and 417 hypomethylated genes. 391 and 467 of total 858 TFs showed hyper‐ or hypomethylation in H_WDvsH_C, respectively. The two cultivars shared 74 common hypermethylated TFs and 91 hypomethylated TFs under water deficit stress (Figure 7c). Previous studies revealed that ‘QG’ and ‘HC’ had different tolerance to drought: ‘QG’ is drought tolerant and ‘HC’ is the opposite. Figure S1 shows the phenotypes of ‘QG’ and ‘HC’ under water deficit, including the photosynthetic capacity, stoma and trichome changes. To explore the possible differentially methylated TFs contributing to the difference in drought tolerance among ‘QG’ and ‘HC’, we analysed the noncommon methylation alterations among Q_WDvsQ_C and H_WDvsH_C. Interestingly, a homeodomain transcription factor, overexpressor of cationic peroxidase 3 (OCP3), presented the most remarkable promoter hypomethylation among all hypomethylation TFs in H_WDvsH_C (Figure 7d and e), but unaltered in ‘QG’ under water deficit (Figure S8). The further expression analysis showed that the *MdOCP3* mRNA abundance was induced in H_WDvsH_C but repressed in Q_WDvsQ_C (Figure 7f). The *Arabidopsis OCP3* referred to an ABA‐dependent negative regulatory factor in drought tolerance in which *ocp3* plants exhibited the enhanced drought resistance (Ramirez *et al*., 2009). Correspondingly, *MdOCP3* was down‐regulated in ‘QG’ (drought‐tolerant) and up‐regulated in ‘HC’ (drought‐sensitive) under water deficit. Also, the expression level of *MdOCP3* in Q_C was much higher than that in H_C (Figure 7f). These results suggested that the differentially expressed *MdOCP3* in ‘QG’ and ‘HC’ under water deficit might contribute to the drought resistance difference between ‘QG’ and ‘HC’. Figure S9 illustrates the six additional representative hypomethylation regions, including *DDB* (*Double B‐BOX 3*) which encodes a homolog of *AtBBX22* (AT1G78600) in *Arabidopsis* and is possibly involved in abiotic stress response; *bHLH*,* WRKY*,* HSP* which encodes a heat shock protein, and *EIN3* which encodes a predicted protein called *Ethylene Insensitive 3* involved in ethylene signalling.

Moreover, the DREB (dehydration‐responsive element binding) transcription factors play an important role in tolerance to water deficit, low temperatures and high‐salt stress (Yang *et al*., 2011). Table S3 displays the methylation differences in DREB genes and ABA‐dependent stress‐induced genes *RD22* in ‘QG’ and ‘HC’ under water deficit. Seven genes were both differentially methylated in Q_WDvsQ_C and H_WDvsH_C. Six genes were only differentially methylated in Q_WDvsQ_C, and nine genes were only differentially methylated in H_WDvsH_C. Indeed, some loci showed different methylation alterations between ‘QG’ and ‘HC’. For example, gene 103413601 encoding a DREB 2F‐like protein was hypomethylated in its promoter region among Q_WDvsQ_C, but unaltered in H_WDvsH_C. Gene 103455074, encoding a DEHYDRATION‐INDUCED 19 homolog 5‐like protein, was hypomethylated in promoter region among Q_WDvsQ_C, but hypermethylated in promoter region among H_WDvsH_C (Table S3).

### Abundant TE genes are hypomethylated under the water deficit stress

**Figure 8:**
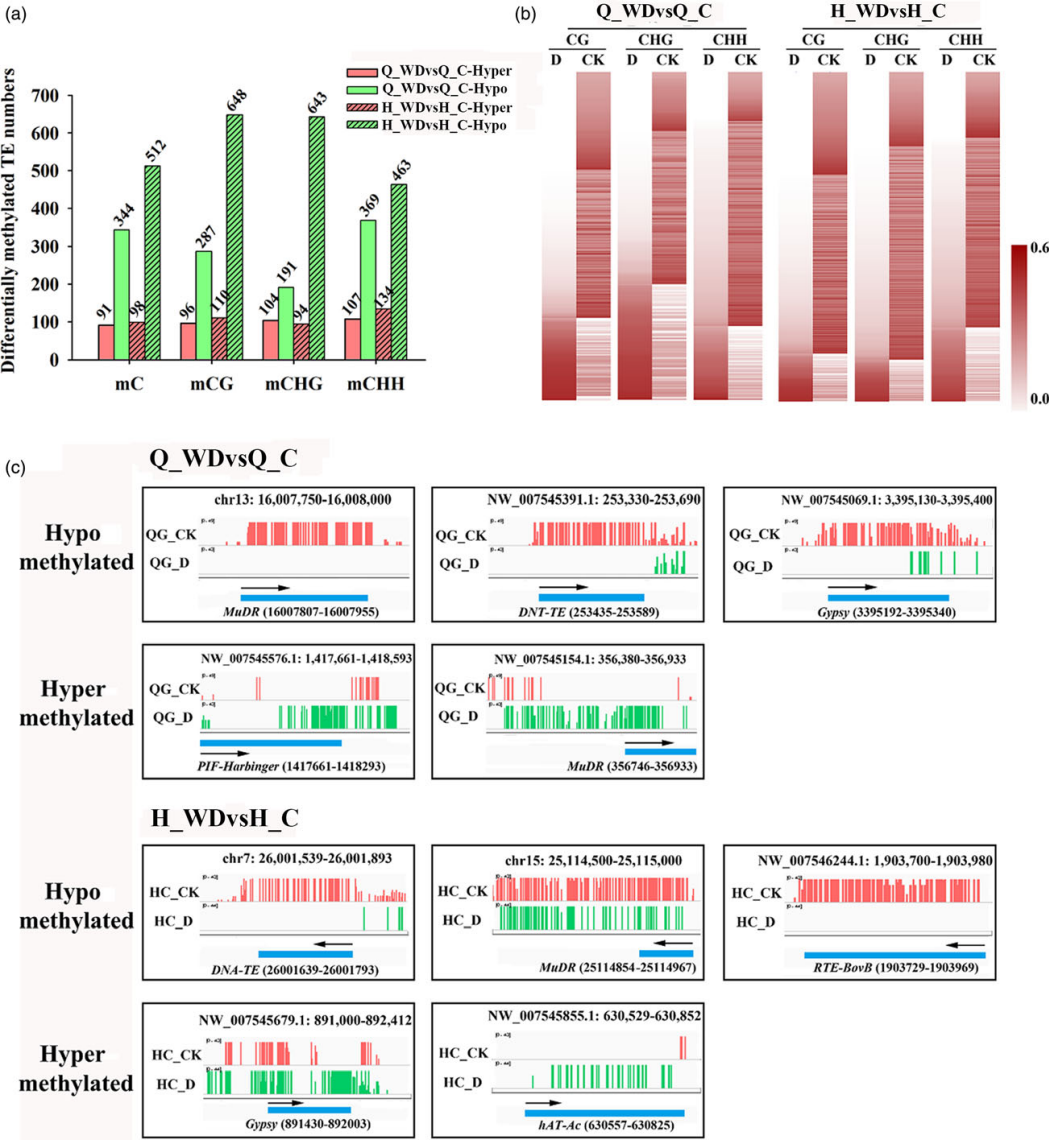
(a) Numbers of differentially methylated TEs in ‘Qinguan’ or ‘Honeycrisp’ under water deficit; (b) heat maps of differentially methylated TEs; (c) IGV snapshots of five representative hypo‐ and hypermethylated TEs in ‘Qinguan’ and ‘Honeycrisp’ under water deficit. Q_WDvsQ_C, ‘Qinguan’ water deficit versus ‘Qinguan’ control; H_WDvsH_C, ‘Honeycrisp’ water deficit versus ‘Honeycrisp’ control.

Previous studies demonstrated that the mobilization and silencing of transposable elements (TE) are often associated with disruption of DNA methylation (Hashida *et al*., 2006; Sahu *et al*., 2013). In both cultivars under water deficit stress, the majority of the differentially methylated TEs were demethylated under water deficit stress (Figure 8a). Based on Fisher's exact test, 344 hypomethylated TEs on total mC sites as well as 91 hypermethylated TEs were identified in cv. ‘Qinguan’ under water deficit stress. For cv. ‘Honeycrisp’, 512 hypomethylated TEs on total mC sites as well as 98 hypermethylated TEs were identified (Figure 8a). For Q_WDvsQ_C, 96, 104 and 107 TEs were hypermethylated in mCG, mCHG and mCHH contexts, respectively, while 287, 191 and 369 TEs were hypomethylated. The combination of H_WDvsH_C showed more differentially methylated TEs than Q_WDvsQ_C, with 110, 94, 134 hypermethylated and 648, 643 and 463 TEs in mCG, mCHG and mCHH sites, respectively (Figure 8a). As shown in Figure 8b, heat maps further exhibited the methylation changes in differentially methylated TEs in mCG, mCHG and mCHH contexts among Q_WDvsQ_C and H_WDvsH_C (Figure 8b). The results revealed that a large proportion of differentially methylated TEs showed demethylation in CG, CHG and CHH contexts. Moreover, H_WDvsH_C combination presented much more hypomethylated TEs in CG and CHG contexts compared with Q_WDvsQ_C (Figure 8b). Previous studies reveal that methylation levels within TEs may dynamically control the expression of transposon genes and the proximal genes in response to stress (Dowen *et al*., 2012). In addition, the demethylation effect on TEs in our study may relate to regulating transposons and genes involved in water deficit response. We selected five representative hypo‐ and hypermethylated TEs in ‘Qinguan’ or ‘Honeycrisp’ under water deficit stress for visualization in detail (Figure 8c).

### Water deficit stress altered methylome patterns combined with differential gene expression

**Figure 9:**
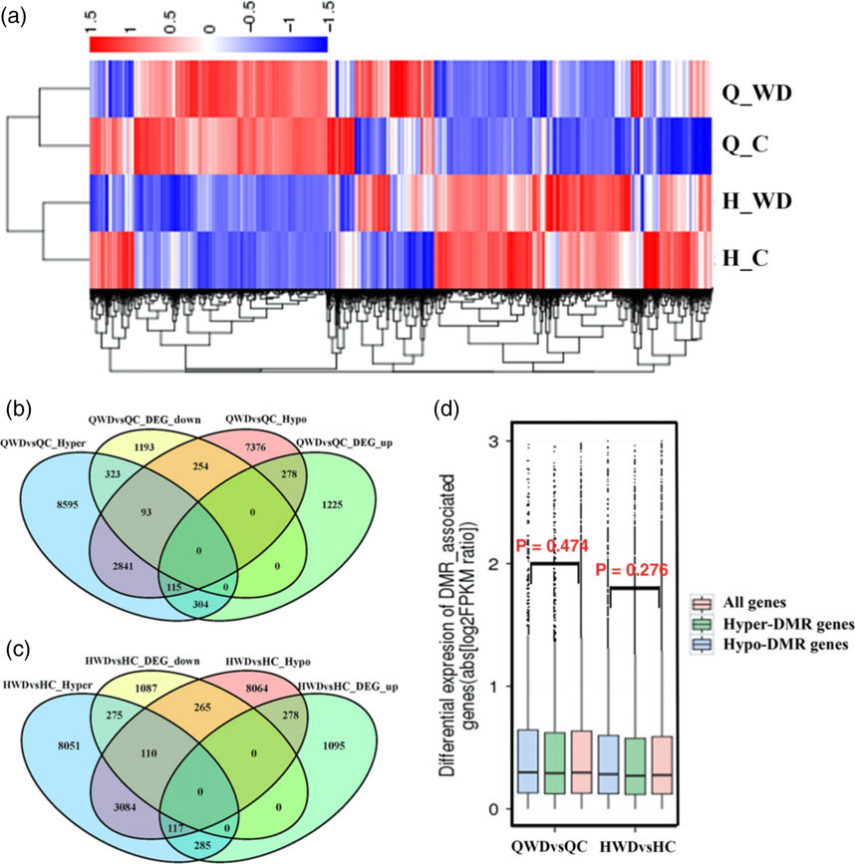
(a) Heap maps of differentially expressed genes (DEGs). Venn diagram of DMGs (differentially methylated genes) and DEGs in QWDvsQC (b) and HWDvsHC (c). (d) Differential expression levels of all genes (red box), hypermethylated genes (green box) and hypomethylated genes (blue box) are displayed as boxplots (boxes represent the quartiles; whiskers mark data within 1.5 interquartile ranges of the quartile; Wilcoxon *P* values are reported). QWDvsQC, ‘Qinguan’ water deficit versus ‘Qinguan’ control; HWDvsHC, ‘Honeycrisp’ water deficit versus ‘Honeycrisp’ control.

To investigate potential transcriptional consequences of widespread methylation changes associated with water deficit stress, we performed mRNA‐seq on the same nonstressed and water deficit‐stressed plants that were used for methylation profiling. In total, 1992 and 1863 genes were up‐regulated and down‐regulated in Q_WDvsQ_C, respectively, while 1775 up‐regulated and 1737 down‐regulated genes were identified in ‘Honeycrisp’ (Figure S10). Hierarchical clustering analysis of genome‐wide differential expression levels is presented in Figure 9a with a heat map. To explore the relationship between methylation changes and transcriptional alterations, we examined the overlap of the DMGs and DEGs. A total of 416 genes were hypermethylated with down‐regulated expression levels and 393 genes were hypomethylated with up‐regulated expression levels in Q_WDvsQ_C. However, 419 up‐regulated and 347 down‐regulated genes were hypermethylated and hypomethylated, respectively. Similarly, in H_WDvsH_C, 385 hyper‐ and 395 hypomethylated genes were negatively correlated with expression changes, while 402 hyper‐ and 375 hypomethylated genes were positively correlated with expression changes (Figure 9b and c). Next, we determined the differential expression levels of all genes or genes associated with hypomethylated or hypermethylated DMRs. As shown in Figure 9d, in both ‘QG’ and ‘HC’ under water deficit stress, although the hypo‐DMR genes (blue box) showed slightly higher expression levels and hyper‐DMR genes (green box) showed slightly lower expression levels compared with all genes (red box), the Wilcoxon *P* values of hypomethylated or hypermethylated genes compared with all genes were not correlated. This indicates that many differential transcript abundances were not associated with the methylation changes. Together, these data suggest that DNA methylation is at least partially responsible for the transcriptional alterations of these genes. A portion of differentially expressed genes is not directly targeted by DNA methylation, but rather is differentially expressed as a consequence of methylation‐dependent changes in transcriptional networks.

### Methylome variations between two apple cultivars under water deficit

**Figure 10:**
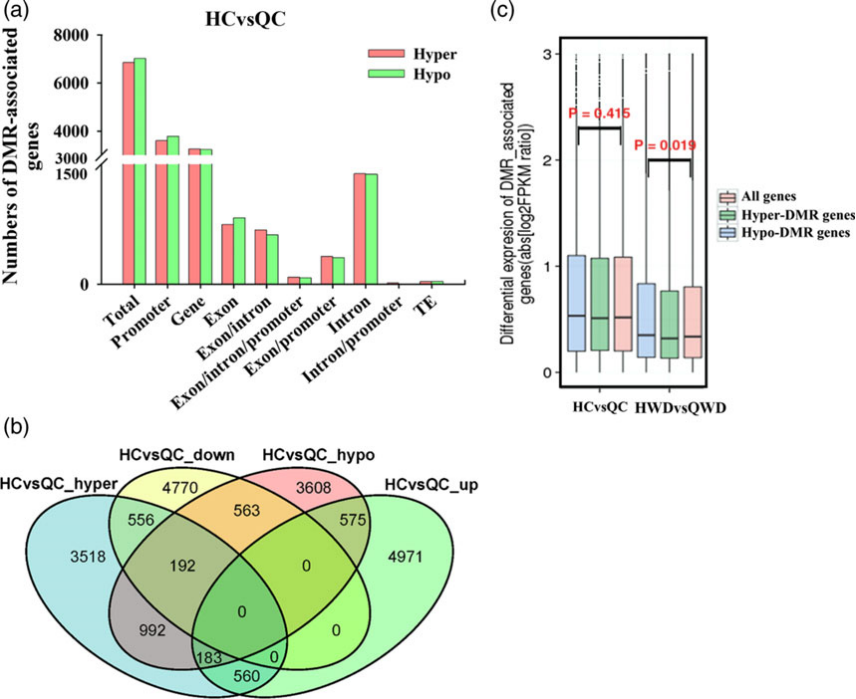
Differential methylation analysis between ‘Qinguan’ (‘QG’) and ‘Honeycrisp’ (‘HC’). (a) Numbers of DMR genes in different gene features among HCvsQC (‘HC’ control versus ‘QG’ control); (b) Venn maps of DMR genes and DEGs; (c) differential expression levels of all genes, hypomethylated genes and hypermethylated genes are displayed as boxplots among HCvsQC and HWDvsQWD (‘HC’ water deficit versus ‘QG’ water deficit; Wilcoxon *P* values are reported).

To investigate the variations in DNA methylation between two apple cultivars, we also identified DMGs in Q_CvsH_C. A total of 3249/3600 hypermethylated and 3232/3784 hypomethylated genes were identified in promoter and gene regions, respectively, leading to a total of 6849 hypermethylated and 7016 hypomethylated genes. A total of 448 and 418 genes were hyper‐ or hypomethylated in both promoter and gene regions. More details are shown in Figure 10a. Combined with the transcriptional data, 748 hypermethylated genes were down‐regulated, while 758 hypomethylated genes were up‐regulated (Figure 10b). Among these genes, 557 (74.5%) and 569 (75.1%) showed at least a twofold change in expression that was also correlated with a change in methylation. Then, we explored the associations of expression levels for all genes or hyper‐ and hypomethylated genes. As shown in Figure 10c, the hypo‐DMR genes (blue box) showed slightly higher expression levels, while hyper‐DMR genes (green box) showed slightly lower expression compared with all genes (red box) among ‘Qinguan’ and ‘Honeycrisp’ control samples, but these were not statistically significant. When we compared Q_WD with H_WD, similar results were observed, as well as hypermethylated genes presented lower expression trends, while hypomethylated genes showed higher expression trends with *P*‐value = 0.019 (Figure 10c).

## Discussion

The ongoing development of high‐throughput sequencing technologies and array‐based methods allows for studying DNA methylation patterns across entire genomes. Whole‐genome bisulphite sequencing (WGBS) is a particularly powerful technology, enabling determination of methylation patterns at single‐nucleotide resolution (Cokus *et al*., 2008; Lister *et al*., 2008). WGBS has been applied to decode an increasing number of plant methylomes, ranging from model plants like *Arabidopsis* to economically important crops like rice (Chodavarapu *et al*., 2012; Zhang *et al*., 2015), maize (Gent *et al*., 2013; Li *et al*., 2014), soya bean (Song *et al*., 2013), tomato (Zhong *et al*., 2013) and also some tree species, such as spruce (*Picea abies*; Ausin *et al*., 2016), oil palm (*Elaeis guineensis*; Ong‐Abdullah *et al*., 2015) and poplar (*Populus Trichocarpa*; Liang *et al*., 2014). Here, we decoded the single‐base apple methylome by WGBS, presenting ~53.6%, ~37.7% and ~8.5% methylation levels at CG, CHG and CHH sequences (Table S2). Based on the previous methylome studies, CG methylation shows the highest levels among the various species, ranging from ~30.5% in *Arabidopsis* to ~92.5% in *Beta vulgaris*. CHG methylation varied from ~9.3% in *Eutrema salsugineum* to ~81.2% in *Beta vulgaris*, and CHH methylation ranged from ~1.1% in *Vitis vinifera* to ~18.8% in *Beta vulgaris* (Niederhuth *et al*., 2016). Previous studies revealed that DNA methylation level is positively correlated with genome size (Ausin *et al*., 2016; Niederhuth *et al*., 2016). The apple methylation levels are moderate among various species, corresponding to the moderate genome size (~742 Mb). Previous studies revealed that hypermethylation was typically found in centromeres and pericentromeric regions and methylation levels showed negative correlation with gene number (Lister *et al*., 2008; Niederhuth *et al*., 2016; Seymour *et al*., 2014). Consistently, our results also showed that CG, CHG and CHH methylation levels are positively correlated with TE density and negatively correlated with gene number (Figure 2). These results suggest that the primary function of DNA methylation is maintenance of genome stability.

Except maintenance of genome stability, the well‐known function of DNA methylation is gene repression (Chan *et al*., 2005). However, an increasing number of genome‐wide methylome analyses demonstrated that the relationship between DNA methylation and transcription is more nuanced than at first realized. Recent rice methylome analysis revealed that promoter methylation repressed gene expression only in heavily methylated gene locus and gene body methylation was usually positively correlated with gene expression (Li *et al*., 2012). A more recent study in *Arabidopsis* presented a more explicit point that DNA methylation only marginally contributed to gene expression (Meng *et al*., 2016). Corresponding to this, our results also did not present an apparent correlation between promoter methylation and gene expression, while gene body methylation appears to be positively correlated with gene expression (Figures 4 and 9d). Although these genome‐scale methylome analyses provide us new viewpoints, abundant DNA methylation studies on specific genes and epialleles indeed reveal the importance of DNA methylation on phenotype regulation and trait heritability, such as *SUPERMAN* (*SUP*) (Jacobsen and Meyerowitz, 1997), *FWA* (Soppe *et al*., 2000) and *Colorless nonripening* (*Cnr*; Manning *et al*., 2006). Together, the primary significance of DNA methylation may be associated with genome stability and TE silencing, which is the normal constituent part of DNA, also called ‘the fifth base’. In most cases, the existence of methylation does not affect gene transcription, but under some certain circumstances, such as environmental interactions, the changes in DNA methylation could alter gene expression and lead to visible phenotypes.

Plant interacts with various environmental factors to survive over their life cycle. Compared with genetic variations, epigenetic alterations are more flexible. Once the environment changes, epigenetics modifications change more easily to adapt to the new environment. Some epigenetic changes are temporary and can be recovered, some of them are heritable, called ‘epigenetic memory’ (Brautigam *et al*., 2013; Mirouze and Paszkowski, 2011; Sahu *et al*., 2013; Vanyushin and Ashapkin, 2011; Verhoeven *et al*., 2010). In our study, profiling the DNA methylomes of apple plants under water deficit stress revealed numerous stress‐induced differentially methylated regions, some of which were associated with gene expression (Figure 9). Similarly, widespread dynamic methylation variations also occurred when *Arabidopsis* plants exposed to pathogen infection (Dowen *et al*., 2012). Indeed, many changes in gene expression were not correlated with the corresponding methylation changes in this study (Figure 9b and c). Similar results were also observed in tomato fruit under chilling stress, revealing that only 37%–67% of different GO term genes presented a negative correlation between methylation level and transcript abundance (Zhang *et al*., 2016). This phenomenon was partly caused by methylation alterations that did not affect gene expression in some regions. On the other hand, abundant gene expression alterations are not directly regulated by DNA methylation, but rather are consequences of methylation‐dependent alterations in transcription networks. This indirect regulation could be mediated by multiple mechanisms. For instance, DNA methylation may turn on the transcripts of genes involved in SA pathway when their expression is required for defence. The increasing SA accumulation could alter the expression levels of abundant downstream genes, implying that DNA methylation could mediate response to biotic stress through the SA signalling (Dowen *et al*., 2012). Our results also showed that abundant methylation alterations were enriched in signalling genes including hormone signalling, implying a similar mechanism. On the other hand, the methylation changes in transcription factors can alter their expression levels, and the misexpressed TFs can further affect the transcripts of their target genes. So, in this case, the transcriptional alterations of downstream TF target genes were dependent on the methylation changes, but not directly controlled by DNA methylation. For example, differential methylation status of the *MYB* promoter contributes to differential anthocyanin accumulation in the peel of apple and pear, resulting in the different peel colour (Telias *et al*., 2011; Wang *et al*., 2013). Paper bagging (environmental factor changes) altered the DNA methylation and histone modifications on *MdMYB1* and activated the *MdMYB1* gene and downstream genes, leading to differential anthocyanin pigmentation (Bai *et al*., 2016). Cold storage of tomato increased methylation of RIN (RIPENING INHIBITOR) and decreased the transcripts of RIN and some of its direct targets (Zhang *et al*., 2016). Compared with regulating structural genes, it is understandable that plants prefer a more energy‐saving and high‐efficient way of controlling TFs to further regulate biological pathways in response to environmental influences. In our study, many TF genes show water deficit‐associated methylation changes, including members of the *WRKY*,* AP2*,* HSP* and *bHLH* classes (Figures 7 and S9), but functional analysis needs to be carried out.

In addition to methylation changes at genes, TEs are also involved in environmental stress adaptions with epigenetic variations (Hashida *et al*., 2006; Sahu *et al*., 2013). We showed that abundant TEs were demethylated under water deficit stress. Consistent with this, global transcriptional analysis of maize TEs in response to abiotic stress revealed that numerous TE genes were up‐regulated and that some of them act as local enhancers to activated stress‐responsive genes (Makarevitch *et al*., 2015). Similarly, methylome analysis in *Arabidopsis* with pathogen infection suggested that methylation levels within TEs may dynamically control the expressions of transposon and the proximal gene in response to stress (Dowen *et al*., 2012). These observations imply that the dynamic DNA demethylation within TEs seen in this study may affect the transcriptional alterations of transposons and proximal genes in response to water deficit stress. Together, comprehensive effects contribute to the final gene expression alterations. DNA methylation changes could affect the gene expression through direct and indirect ways. Those gene expression alterations indirectly regulated by DNA methylation are possibly the consequences of methylation‐dependent alterations in transcription networks. Our genome‐wide approach uncovered unique aspects of water deficit‐induced methylome changes in apple. These data will be useful for studying methylation regulation in response to abiotic stress.

## Materials and methods

### Plant material and stress treatment

‘Qinguan’ (‘QG’) and ‘Honeycrisp’ (‘HC’) plants were cultivated and water deficit stress treatments were performed as described in a previous study (Zhou *et al*., 2015). The experiments were conducted at Northwest A&F University, Yangling, located in loess plateau of China (34°20′N, 108°24′E). All plants were grown in a glasshouse under ambient light, at 20–25 °C and 50%–75% relative humidity. At the end of May, uniform trees of each cultivar (100 trees each) were divided into two equal groups and subjected to the following treatments: (i) well watered, irrigated daily to maintain 75%–85% field capacity (FC) and (ii) moderate water deficit, irrigated daily to achieve 45%–55% FC. These treatments continued for 3 months. At the end of treatments, 30 representative plants were selected, and leaves at similar node number and developmental stage from plants from each treatment were collected and frozen in liquid nitrogen for −80 °C storage. Based on the treatments, sample were designated as follows: Q_C, well‐watered ‘QG’; Q_WD, moderately water deficit‐stressed ‘QG’; H_C, well‐watered ‘HC’; H_WD, moderately water deficit‐stressed ‘HC’ (Figure S1). Net photosynthesis rate (Pn), transpiration rate (Tr), stomatal conductance (Gs) and instantaneous water‐use efficiency (WUEi) were measured by the ALI‐Cor 6400 portable photosynthesis system (LI‐COR, Huntington Beach, CA) on sunny days during morning hours, 9–11 AM. 10 fully expanded mature leaves per treatment were tested from different trees. For scanning electron microscopy (SEM) analysis, four leaves (from the fifth leaf position on each plant) were sampled per genotype per treatment. Samples were immediately fixed with the 4% glutaraldehyde solution in 0.1 m phosphate‐buffered saline (PBS, pH 6.8). Then, samples were rinsed with PBS for five times and dehydrated in the graded ethanol series, vacuum‐dried and gold‐coated. JSM‐6360LV microscope (JEOL Ltd., Tokyo, Japan) was applied for SEM analysis (Figure S1).

### DNA extraction and BS‐seq library construction

Total genomic DNA was extracted from leaves according to a modified CTAB DNA extraction protocol (Kobayashi *et al*., 1998). A total amount of 5.2 μg genomic DNA spiked with 26 ng lambda DNA was fragmented by sonication to 200–300 bp with a Covaris S220 sonicator, followed by end repair and adenylation. Lambda DNA was used as an unmethylated control for calculating the bisulphite conversion rate. Then, the DNA fragments were treated twice with bisulphite using the EZ DNA Methylation‐Gold™ Kit (Zymo Research). BS‐seq library constructions and sequencing utilized two biological replicates per sample. Libraries were sequenced on the Illumina Hiseq 2500 platform (Novogene, Beijing, China).

### Reads mapping and DMR analysis

Bismark software (version 0.12.5; Krueger and Andrews, 2011) was used to perform alignments of bisulphite‐treated reads to the reference genome (https://www.ncbi.nlm.nih.gov/genome/358?genome_assembly_id=28726) using default parameters. The reference genome was converted into a bisulphite‐converted version (C to T and G to A converted) and then indexed using Bowtie2 (Langmead and Salzberg, 2012). Sequence reads were also transformed into fully bisulphite‐converted versions (C to T and G to A converted) before reads were aligned to similarly converted versions of the genome in a directional manner. Sliding‐window approach was applied for methylation‐level analysis. With window size = 3000 bp and step size = 600 bp (Smallwood *et al*., 2014), the sum of methylated and unmethylated read counts were calculated in each window. Methylation level (ML) for each C site shows the fraction of methylated Cs. It is defined as: ML (C) = reads (mC)/reads (mC) + reads (C). Calculated ML was further corrected with the bisulphite nonconversion rate according to previous studies (Lister *et al*., 2013). Given the bisulphite nonconversion rate *r*, the corrected ML was estimated as: ML (corrected) = ML−*r*/1−*r*. The percentage of methylation levels was calculated as the proportion of mCs on the total C sites (Table S2). The relative proportion of mCs in three contexts was calculated as the proportion of mCG, mCHG and mCHH on the total mC sites, respectively (Figure 2a). Differentially methylated regions (DMRs) were identified by swDMR software (http://122.228.158.106/swDMR/), which applied a sliding‐window approach. The window was 1000 bp and step length was 100 bp. The Fisher test was applied to detect significant DMRs. After DMRs were identified, genes located in DMRs were characterized. Then, DMR‐associated genes were analysed for Gene Ontology (GO) and KEGG enrichment. GO analysis was implemented with the GOseq R package (Young *et al*., 2010), which corrects for gene length bias. GO terms with corrected *P*‐value less than 0.05 were considered significantly enriched by DMR‐related genes. Kyoto Encyclopedia of Genes and Genomes (KEGG: http://www.genome.jp/kegg/) was applied for understanding the pathway enrichment of DMR genes (Kanehisa *et al*., 2008). KOBAS software was used to test the statistical enrichment of DMR‐related genes in KEGG pathways (Mao *et al*., 2005). Also, the differentially methylated genes were assigned to different functional bins based on MapMan software (Thimm *et al*., 2004).

### Transposable elements (TEs) characterization and analysis

TEs were screened and annotated using the RepeatMasker program (http://www.repeatmasker.org/). The parameters were set as follows: match:2; mismatch: 7; delta: 7; PM:80; PI:10; minscore: 50; maximum period: 500. Based on the RepeatMasker annotation, the top 10 types of TEs in terms of abundance were analysed for methylation levels. We used Fisher's exact test to identify the differentially methylated TEs. The FDR (false discovery rate) method was applied for multiple testing adjustment. After statistical testing, differentially methylated TEs were defined with two criteria: *P*‐value <0.05 and differential methylation level >2‐fold.

### RNA extraction and transcriptome sequencing

The plant materials used for transcriptome analysis were the same as the materials applied in methylome analysis. Three biological replicates were used for transcriptome analysis, and two biological replicates were used for methylome. Total RNAs were extracted by RNAprep Pure Plant Kit (DP441, TIANGEN Biotech), following the manufacturer's instructions. RNA quality and integrity were tested using NanoPhotometer spectrophotometer (IMPLEN, Westlake Village, CA) and RNA Nano 6000 Assay Kit of the Bioanalyzer 2100 system (Agilent Technologies, Santa Clara, CA). A total amount of 1 μg qualified RNA per sample was used as input for the RNA sample preparations. Sequencing libraries were generated using NEBNext^®^ Ultra™ RNA Library Prep Kit for Illumina^®^ (NEB, Ipswich, MA), following the manufacturer's recommendations and instructions. The library fragments were purified with AMPure XP system (Beckman Coulter, Beverly, MA) to select cDNA fragments of preferentially 250–300 bp in length. Clustering of index‐coded samples was performed on cBot Cluster Generation System by TruSeq PE Cluster Kit v3‐cBot‐HS (Illumina). After filtrating the adapter and low‐quality reads, clean reads were aligned to apple reference genome (https://www.ncbi.nlm.nih.gov/genome/358?genome_assembly_id=28726) by TopHat v2.0.12, in which the parameter was set with mismatch=2 (Kim *et al*., 2013). HTSeq v0.6.1 was used to count the reads numbers mapped to each gene (Anders *et al*., 2015), then the FPKM (fragments per kilobase of transcript sequence per millions base pairs sequenced) of each gene was calculated according to their length and reads count. DESeq R package (v1.18.0) was applied for differential gene expression analysis (Anders and Huber, 2012). The resulting *P*‐values were adjusted by Benjamini and Hochberg’ approach for controlling false discovery rate. Genes with adjusted *P*‐value <0.05 were considered as differentially expressed. The differentially expressed genes were further performed with Gene Ontology (GO) and KEGG analysis (KEGG: http://www.genome.jp/kegg/). GOseq R package was applied for the GO enrichment analysis (Young *et al*., 2010), and KOBAS software was used to test the statistical enrichment of differentially expressed genes in KEGG pathway (Mao *et al*., 2005).

### Identification of apple DNA methylation‐related genes and real‐time PCR analysis

*Arabidopsis* DNA methylation‐related protein sequences were used as queries for NCBI blastp program. The accession numbers of *Arabidopsis* and apple DNA methylation‐related proteins are listed in Table S1. ‘Golden Delicious’ plant materials were used for qRT‐PCR analysis: buds of ‘Golden Delicious’ grafted onto the uniform apomixis rootstock *M. hupehensis* Rehd. var. Pingyiensis. Seedlings were grown in plastic pots with the same soil weight and irrigation. Five‐month‐old uniform plants were treated with drought treatment, and leaves were harvested after 0, 2, 4, 6 and 8 days. Total RNA was extracted using cetyltrimethyl ammonium bromide (CTAB) method (Chang, 1993). A total of 1.5 μg RNA was used for first‐strand complementary DNA (cDNA) synthesis using the RevertAid first‐strand cDNA synthesis kit (ThermoFisher K1622, Waltham, MA, USA) according to the manufacturer's instructions. The primers were diluted in GoTaq^®^ qPCR Master Mix (A6001; Promega, Madison, WI, USA), and the amplification mixture volume was 10 μL per reaction. Reaction conditions were performed using an initial incubation for 2 min at 95 °C and then cycled at 95 °C/15 s and 58 °C/15 s followed by 72 °C/45 s for 40 cycles. Reactions were run on the StepOnePlus™ Real‐Time PCR System (ThermoFisher). Each sample was performed with three replicates and two technical replicates. The untreated samples were used as the calibrator for expression data analysis. Transcripts of *Malus* elongation factor 1 alpha gene (EF‐1α, DQ341381) were used as an endogenous control, and the comparative *C*_t_ method $2^{-\Delta \Delta C_{t}}$was adopted to calculate the expression data. The primers for real‐time PCR are listed in Table S4.

## Conflict of interest

The authors confirm no conflict of interest.

## Availability of data and materials

The methylome data have been deposited into the NCBI Short Read Archive (SRA, https://www.ncbi.nlm.nih.gov/sra/) under accession number SRP097376.

## Supporting information


**Figure S1** The phenotype of ‘Qinguan’ (‘QG’) and ‘Honeycrisp’ (‘HC’) plants under water deficit stress. ‘CK’ represented well‐watered plants and ‘D’ represented moderately water deficit plants.
**Figure S2** Methylation landscapes of ‘Honeycrisp’ genome.
**Figure S3** (a) Methylation levels for each of the eight samples. The *Y*‐axis represents methylation levels (10 Kb/bin) and the width of each violin represents mC abundance at the corresponding methylation level; (b) The methylation density of each of the eight samples. The *Y*‐axis represents the percentage of mC density among the total cytosine sites (10 Kb/bin). The width of each violin represents the mC abundance at the corresponding methylation density. QG_CK represents ‘Qinguan’ at control conditions; QG_D represents ‘Qinguan’ under water deficit treatment; HC_CK represents ‘Honeycrisp’ at control conditions, HC_D represents ‘Honeycrisp’ under water deficit stress. 1 and 2 in CK1, CK2, D1, D2 denote corresponding replicates.
**Figure S4** Distribution of mCs identified on the sense and antisense strands of ‘Qinguan’ (‘QG’) and ‘Honeycrisp’ (‘HC’) chromosomes (a) and mC densities in CG, CHG, and CHH sequence contexts in each chromosomes (b) under control conditions (CK). The green, purple and yellow lines represent mC densities in CG, CHG, and CHH contexts, respectively.
**Figure S5** GO (Gene Ontology) enrichment analysis of methylated and unmethylated genes in ‘Qinguan’ (‘QG’) or ‘Honeycrisp’ (‘HC’) apple varieties under control conditions (CK).
**Figure S6** Boxplots of DMR (Differentially Methylated Region) length on each chromosome and DMR methylation levels in ‘Qinguan’ (a) or ‘Honeycrisp’ (b) in response to water deficit stress. QG_CK represents ‘Qinguan’ at control conditions; QG_D represents ‘Qinguan’ under water deficit treatment; HC_CK represents ‘Honeycrisp’ at control conditions, HC_D represents ‘Honeycrisp’ under water deficit stress.
**Figure S7** MapMan analysis of DMR‐associated genes involved in ubiquitin mediated proteolysis pathway in ‘Qinguan’ or ‘Honeycrisp’ in response to water deficit stress.
**Figure S8** The IGV snapshot of methylation levels in *MdOCP3* promoter region among Q_WDvsQ_C (‘Qinguan’ water deficit versus ‘Qinguan’ control).
**Figure S9** IGV snapshots of six differentially methylated transcriptional factors (TFs).
**Figure S10** Differentially expressed genes (DEGs) in ‘Qinguan’ or ‘Honeycrisp’ in response to water deficit stress.

**Table S1** Homologs of DNA methyltransferase and demethylase proteins in apple.
**Table S2** Percentage of methylation levels of ‘Qinguan’ and ‘Honeycrisp’.
**Table S3** Methylation differences of dehydration‐related genes in ‘Qinguan’ and ‘Honeycrisp’ under water deficit.
**Table S4** Primers used for qRT‐PCR in this study.
