# Supplementary material for: Single‐base methylome analysis reveals dynamic epigenomic differences associated with water deficit in apple
Source: Plant Biotechnol J. 2017 Sep 22;16(2):672–87. doi: 10.1111/pbi.12820 (PMC5787839; doi:10.1111/pbi.12820)
Supplement: Supplementary file 1 — Figure S1 The phenotype of ‘Qinguan’ (‘QG’) and ‘Honeycrisp’ (‘HC’) plants under water deficit stress. ‘CK’ represented well‐watered plants and ‘D’ represented moderately water deficit plants. Figure S2 Methylation landscapes of ‘Honeycrisp’ genome. Figure S3 (a) Methylation levels for each of the eight samples. The Y‐axis represents methylation levels (10 Kb/bin) and the width of each violin represents mC abundance at the corresponding methylation level; (b) The methylation density of each of the eight samples. The Y‐axis represents the percentage of mC density among the total cytosine sites (10 Kb/bin). The width of each violin represents the mC abundance at the corresponding methylation density. QG_CK represents ‘Qinguan’ at control conditions; QG_D represents ‘Qinguan’ under water deficit treatment; HC_CK represents ‘Honeycrisp’ at control conditions, HC_D represents ‘Honeycrisp’ under water deficit stress. 1 and 2 in CK1, CK2, D1, D2 denote corresponding replicates. Figure S4 Distribution of mCs identified on the sense and antisense strands of ‘Qinguan’ (‘QG’) and ‘Honeycrisp’ (‘HC’) chromosomes (a) and mC densities in CG, CHG, and CHH sequence contexts in each chromosomes (b) under control conditions (CK). The green, purple and yellow lines represent mC densities in CG, CHG, and CHH contexts, respectively. Figure S5 GO (Gene Ontology) enrichment analysis of methylated and unmethylated genes in ‘Qinguan’ (‘QG’) or ‘Honeycrisp’ (‘HC’) apple varieties under control conditions (CK). Figure S6 Boxplots of DMR (Differentially Methylated Region) length on each chromosome and DMR methylation levels in ‘Qinguan’ (a) or ‘Honeycrisp’ (b) in response to water deficit stress. QG_CK represents ‘Qinguan’ at control conditions; QG_D represents ‘Qinguan’ under water deficit treatment; HC_CK represents ‘Honeycrisp’ at control conditions, HC_D represents ‘Honeycrisp’ under water deficit stress. Figure S7 MapMan analysis of DMR‐associated genes involved in ubiquitin mediated prot [file PBI-16-672-s001.docx]

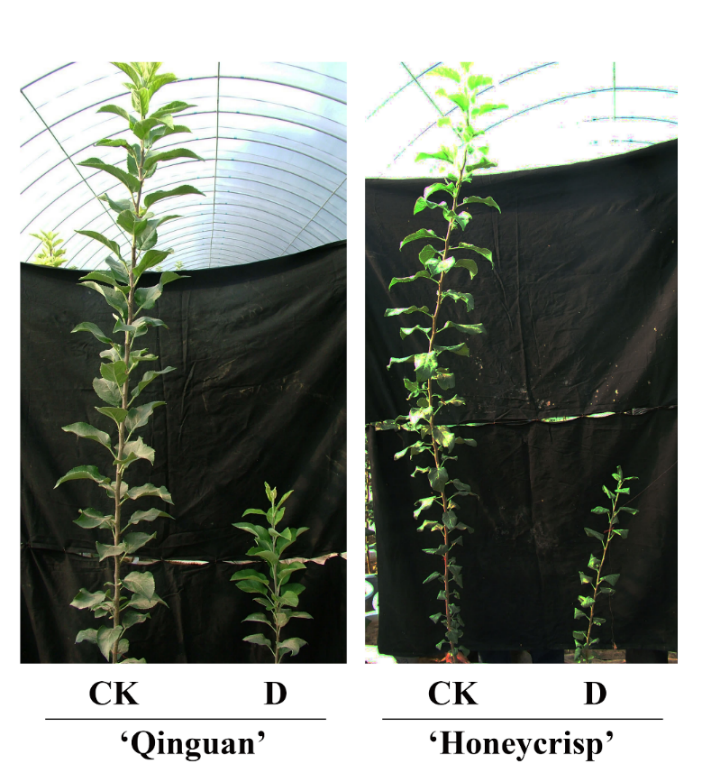


**A**


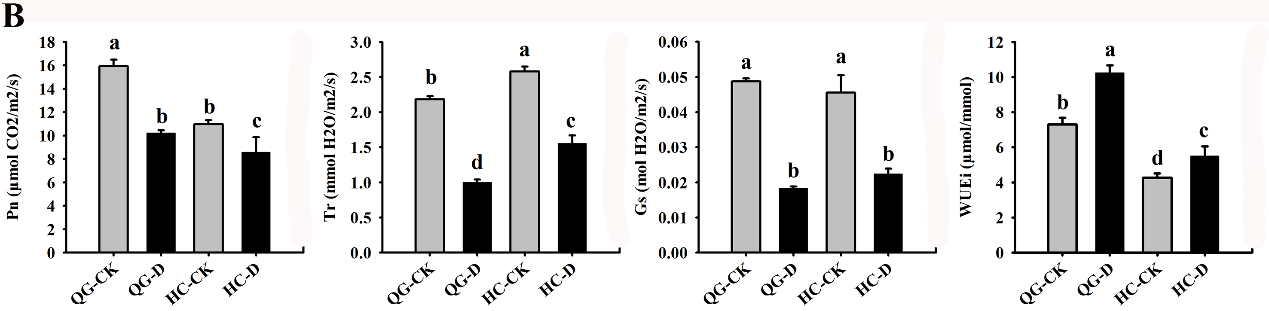


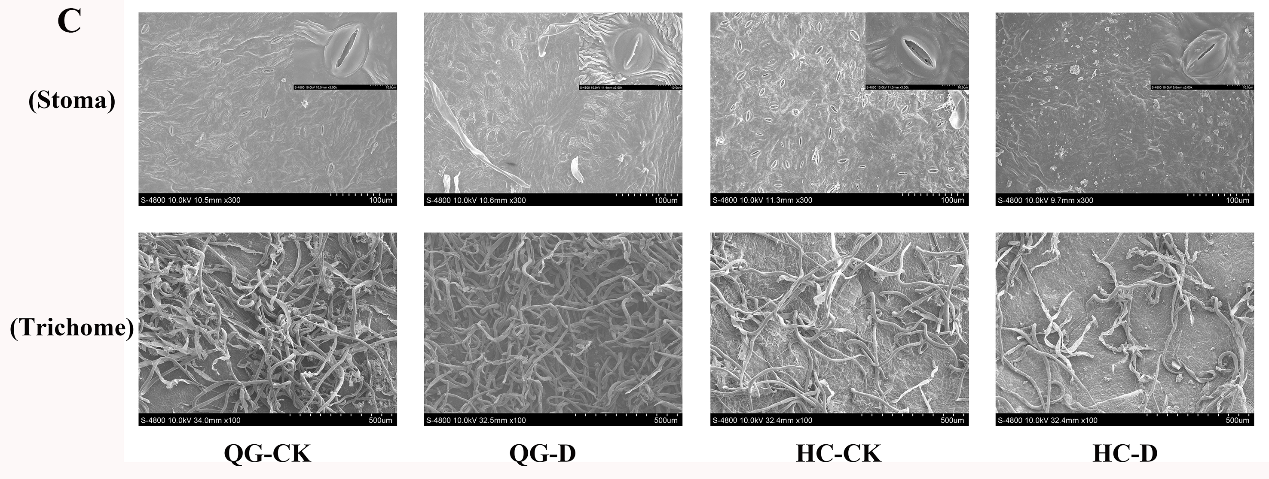


**Fig. S1** The phenotype of ‘Qinguan’ (‘QG’) and ‘Honeycrisp’ (‘HC’) plants under water-deficit stress. “CK” represented well-watered plants and “D” represented moderately water-deficit plants. (A) The phenotypes of ‘QG’ and ‘HC’ plants under water-deficit stress. (B) Gas exchange parameters of ‘QG’ and ‘HC’ plants leaves. Pn, Net photosynthesis rate; Tr, Transpiration rate; Gs, Stomatal conductance; WUEi, instantaneous water-use efficiency. (C) The stoma and trichome of ‘QG’ and ‘HC’ plants under water deficit stress.

**
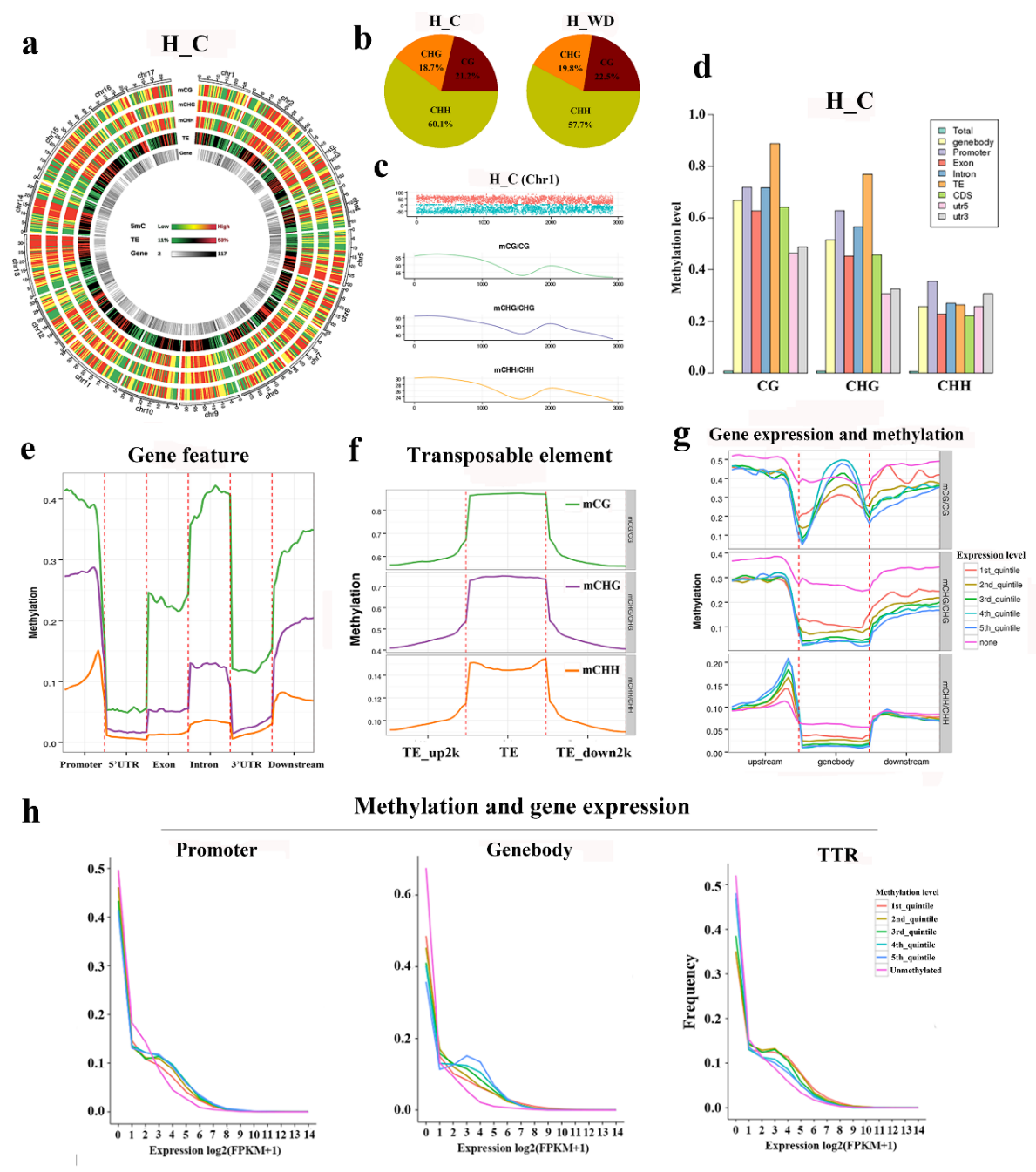
**

**Fig. S2 Methylation landscapes of ‘Honeycrisp’ genome.** (a) Circos plots of chromosomes in apple genome. Track order: Density plot of 5mC in CG, CHG and CHH context; density of transposable elements (TEs); gene density of each chromosome. (b) Relative proportions of mCs in three sequence contexts (CG, CHG and CHH) in ‘Honeycrisp’. (c) Distributions of 5-methylcytosine density on chromosome 1. (d) Percentage of methylation levels of promoter, transposable element (TE), coding genes with 5’UTR, exon, intron, CDS, 3’UTR regions. (e) Distributions of DNA methylation levels among gene features, including promoter, 5’UTR, Exon, Intron, 3’UTR, and downstream 2kb. (f) Percentage of methylation levels among TE regions and their 2 kb upstream and downstream regions. (g) Distributions of methylation levels within gene bodies partitioned by different expression levels: 1^st^_quintile is the lowest and 5^th^_quintile is the highest; genes with FPKM value <0.1 were considered non-expressed (none). (h) Expression profiles of methylated genes compared with unmethylated genes. Methylated genes were further divided into quintiles based on promoter, gene body and Transcriptional Termination Region (TTR) methylation levels: 1^st^_quintile is the lowest and 5^th^_quintile is the highest.

**A** **B**


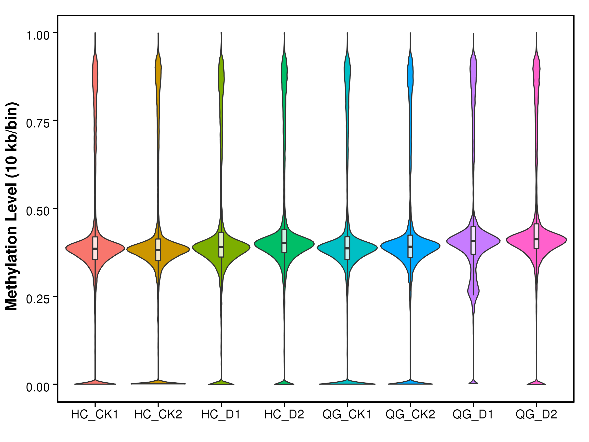

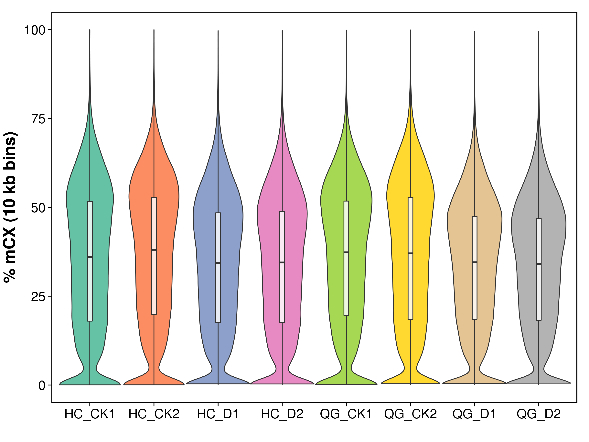


**Fig. S3** (A) Methylation levels for each of the eight samples. The Y-axis represents methylation levels (10 Kb/bin) and the width of each violin represents mC abundance at the corresponding methylation level; (B) The methylation density of each of the eight samples. The Y-axis represents the percentage of mC density among the total cytosine sites (10 Kb/bin). The width of each violin represents the mC abundance at the corresponding methylation density. QG_CK represents ‘Qinguan’ at control conditions; QG_D represents ‘Qinguan’ under water deficit treatment; HC_CK represents ‘Honeycrisp’ at control conditions, HC_D represents ‘Honeycrisp’ under water deficit stress. 1 and 2 in CK1, CK2, D1, D2 denote corresponding replicates.

**a**


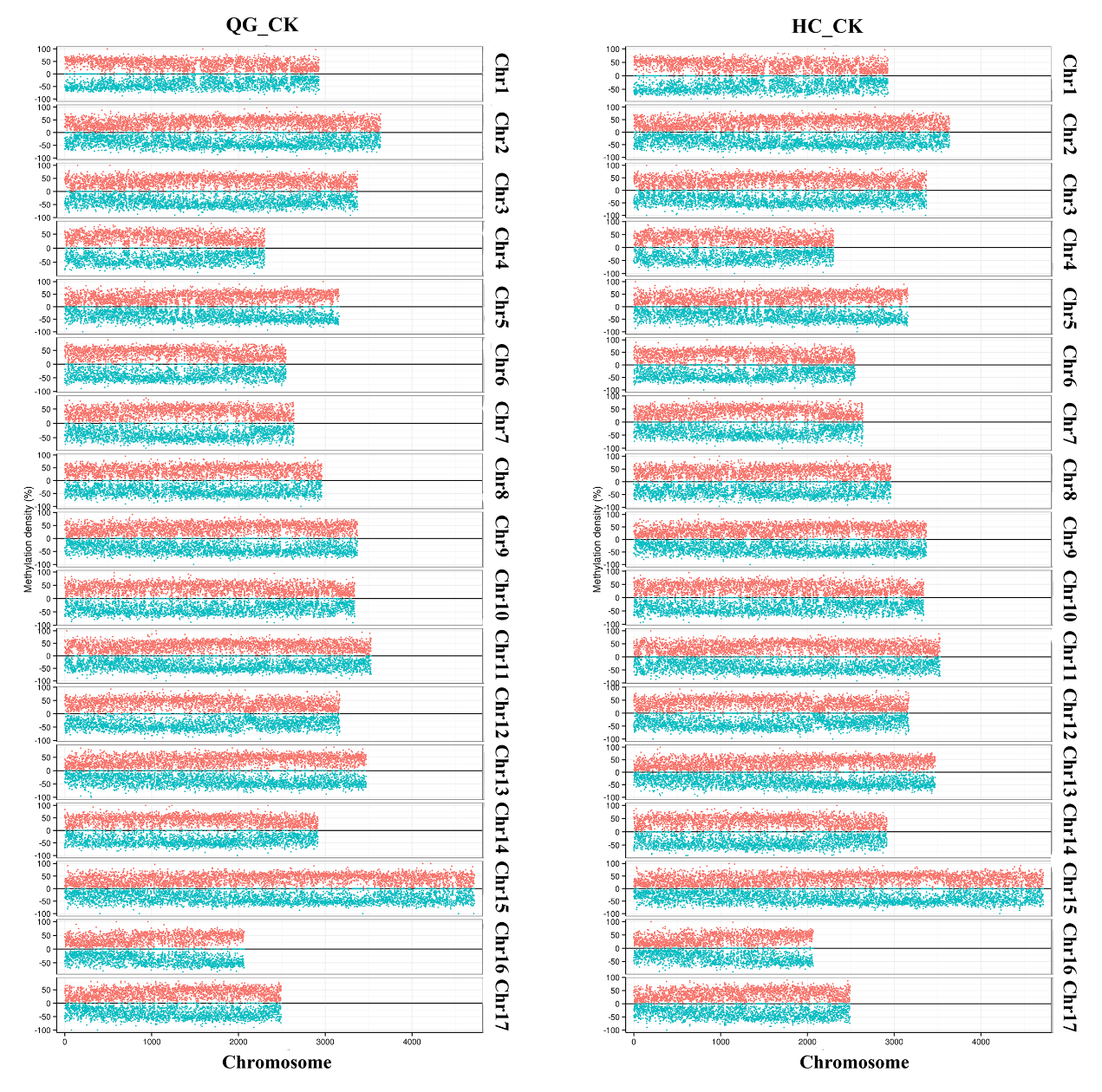


**b**


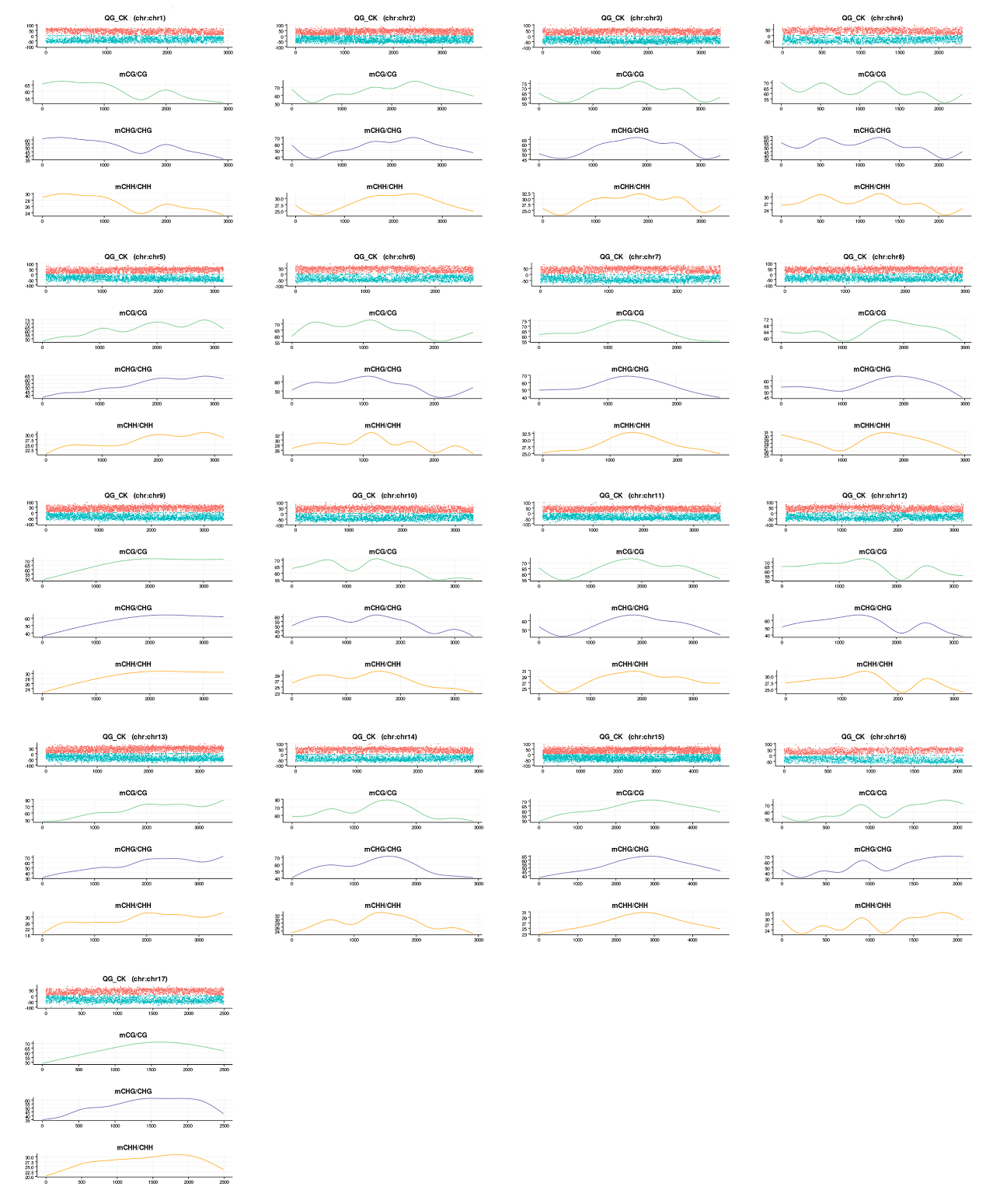


**
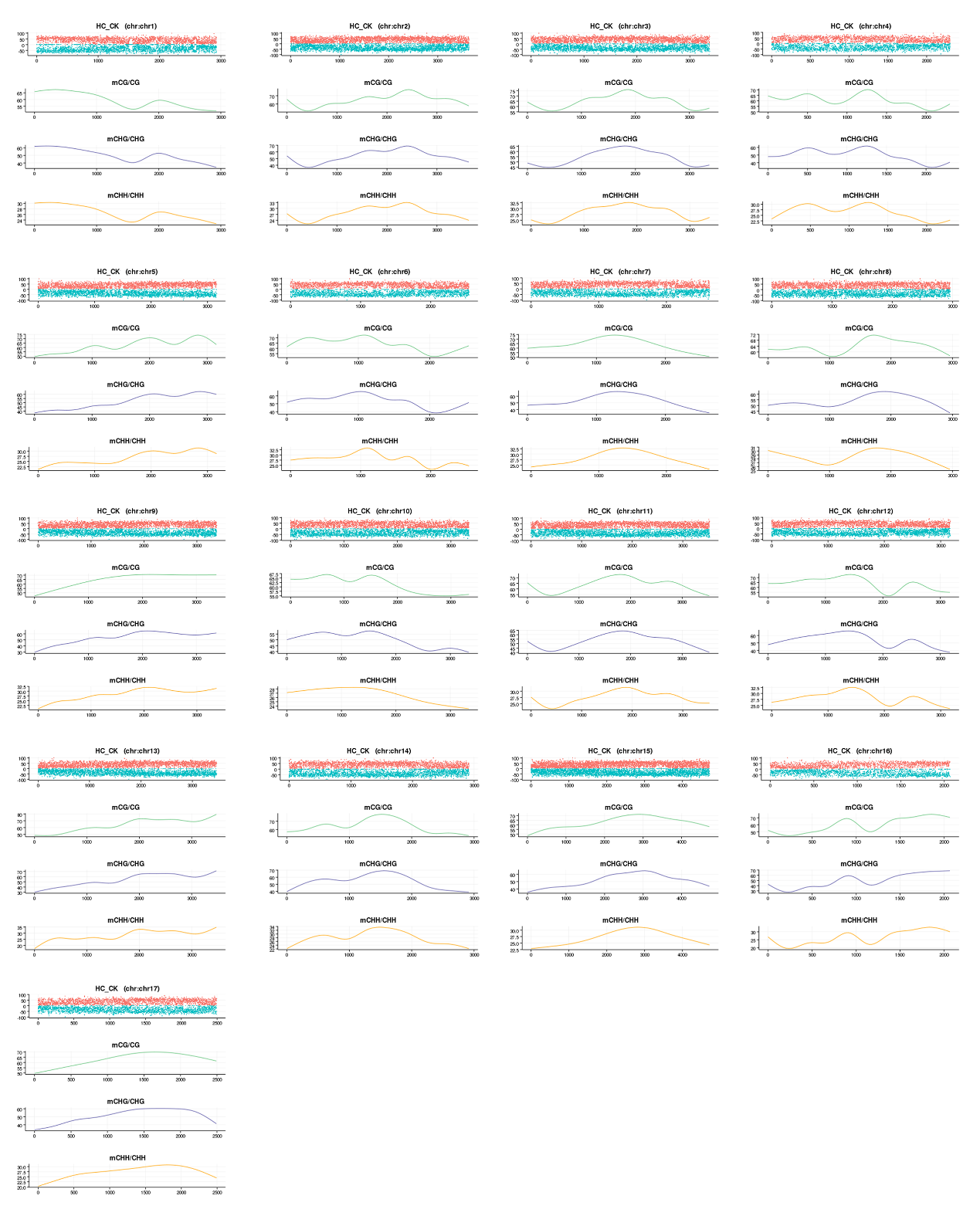
**

**Fig. S4** Distribution of mCs identified on the sense and antisense strands of ‘Qinguan’ (‘QG’) and ‘Honeycrisp’ (‘HC’) chromosomes (**a**) and mC densities in CG, CHG, and CHH sequence contexts in each chromosomes (**b**) under control conditions (CK). The green, purple and yellow lines represent mC densities in CG, CHG, and CHH contexts, respectively.


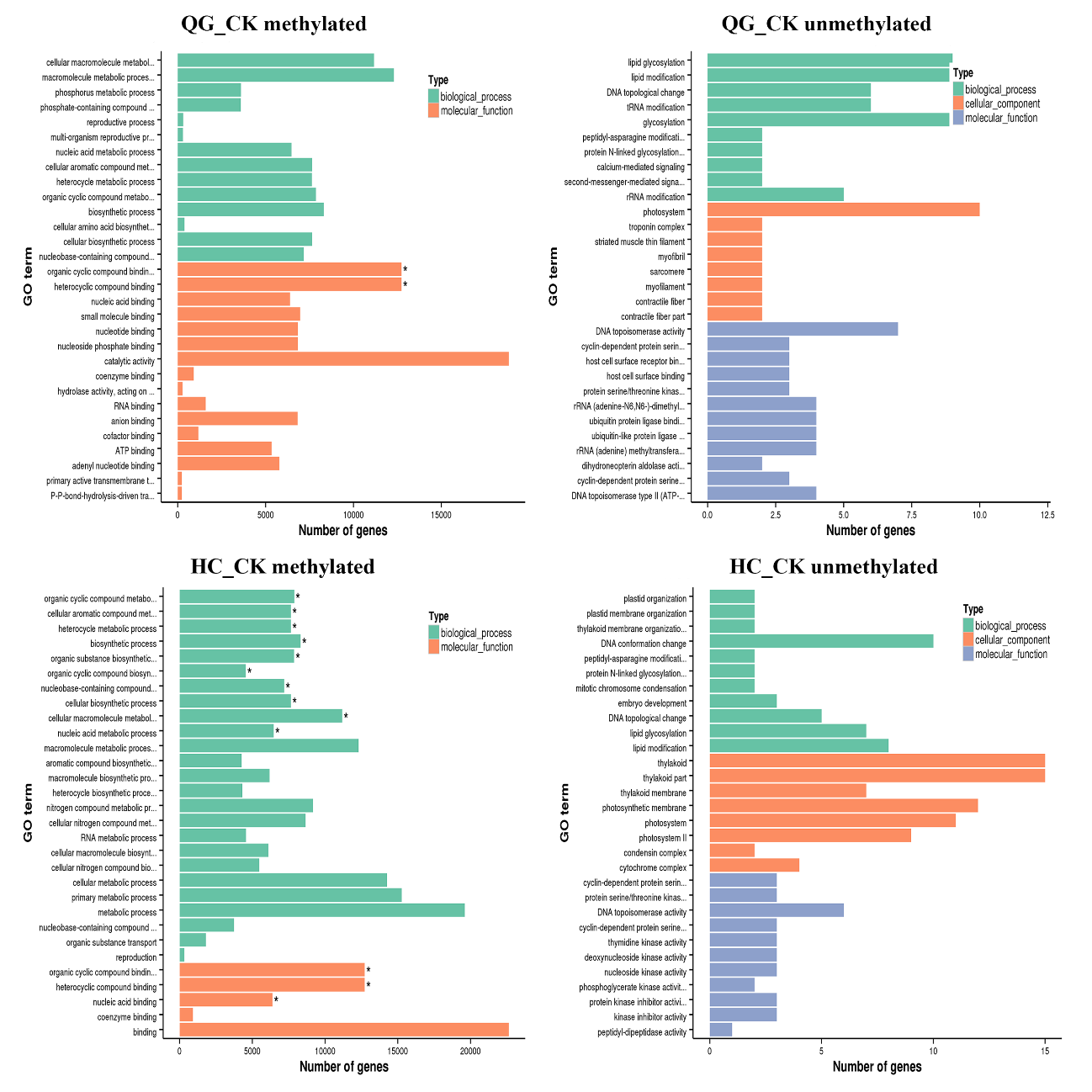


**Fig. S5** GO (Gene Ontology) enrichment analysis of methylated and unmethylated genes in ‘Qinguan’ (‘QG’) or ‘Honeycrisp’ (‘HC’) apple varieties under control conditions (CK).


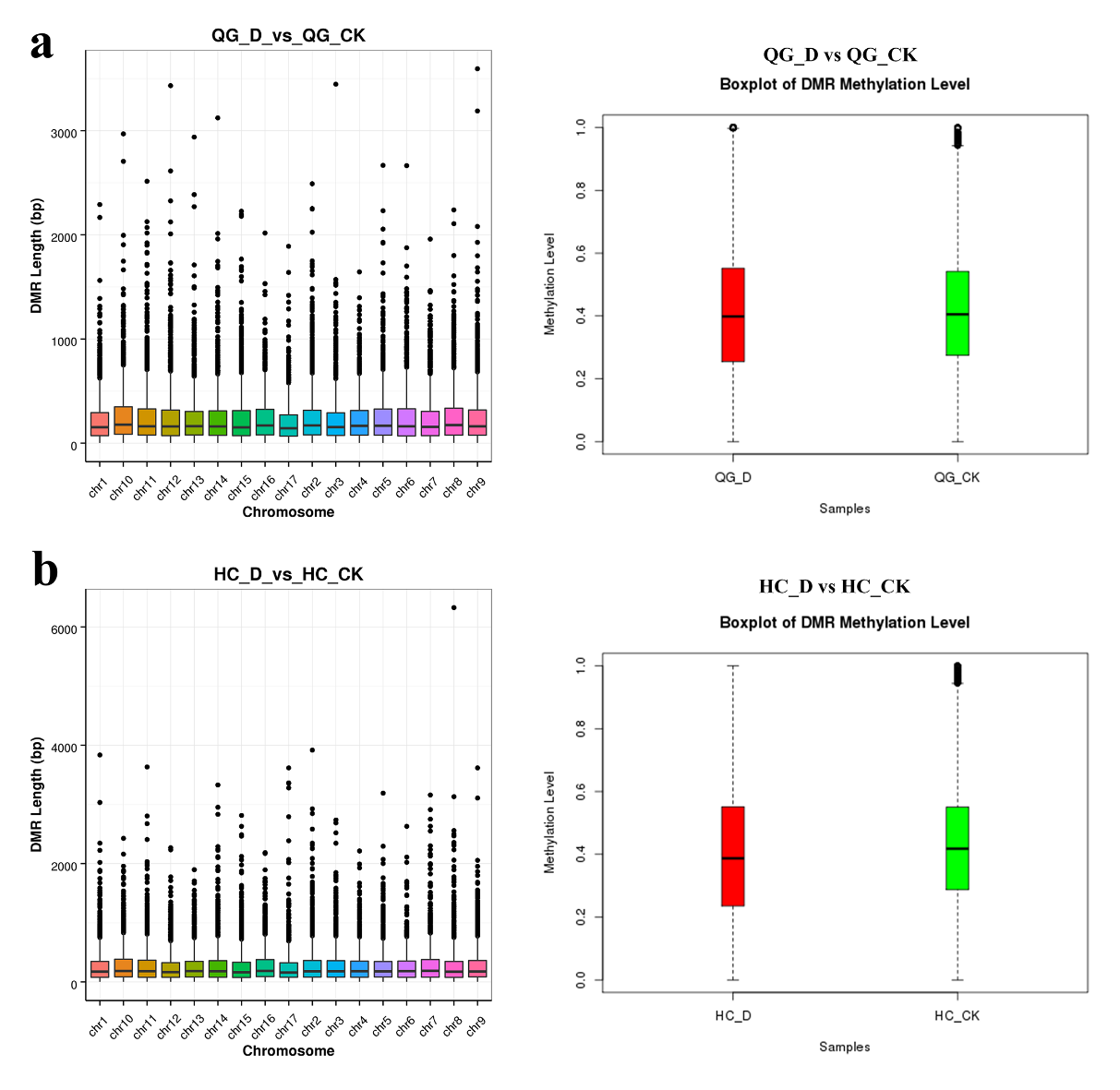


**Fig. S6** Boxplots of DMR (Differentially Methylated Region) length on each chromosome and DMR methylation levels in ‘Qinguan’ (a) or ‘Honeycrisp’ (b) in response to water deficit stress. QG_CK represents ‘Qinguan’ at control conditions; QG_D represents ‘Qinguan’ under water deficit treatment; HC_CK represents ‘Honeycrisp’ at control conditions, HC_D represents ‘Honeycrisp’ under water deficit stress.


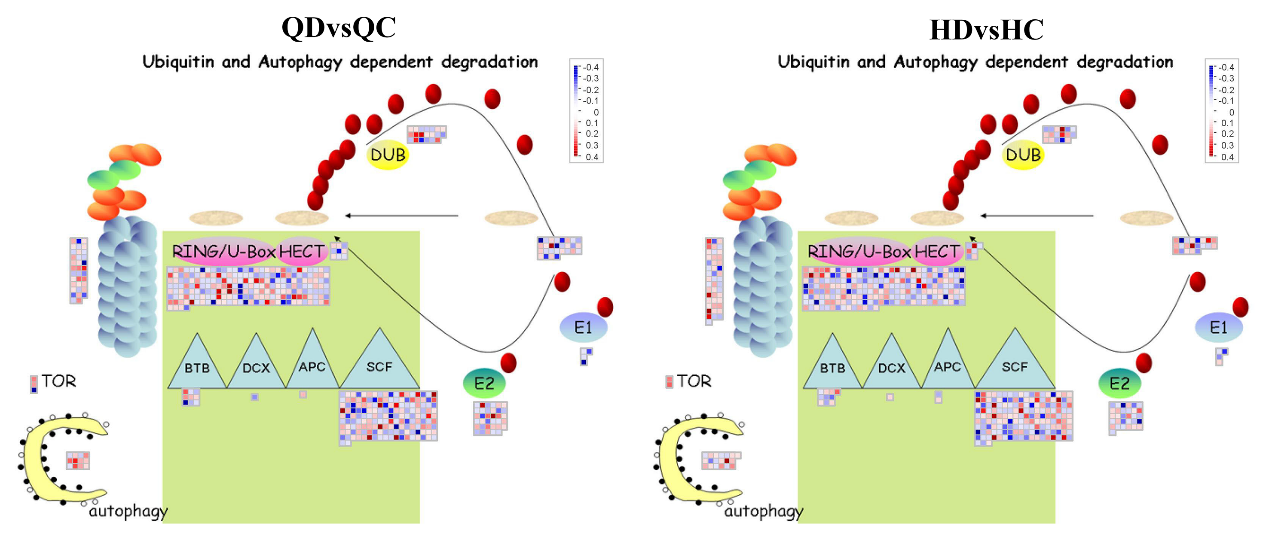


**Fig. S7** MapMan analysis of DMR-associated genes involved in ubiquitin mediated proteolysis pathway in ‘Qinguan’ or ‘Honeycrisp’ in response to water deficit stress. QC represents ‘Qinguan’ at control conditions; QD represents ‘Qinguan’ under water deficit treatment; HC represents ‘Honeycrisp’ at control conditions, HD represents ‘Honeycrisp’ under water deficit stress.


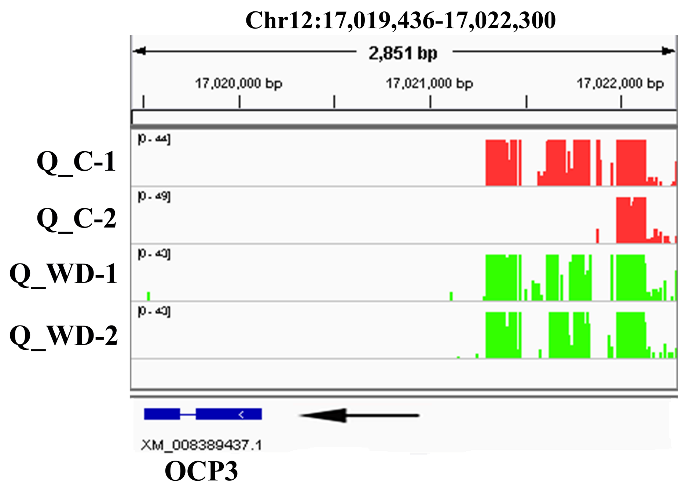


**Fig. S8** The IGV snapshot of methylation levels in *MdOCP3* promoter region among Q_WDvsQ_C (‘Qinguan’ water-deficit versus ‘Qinguan’ control).


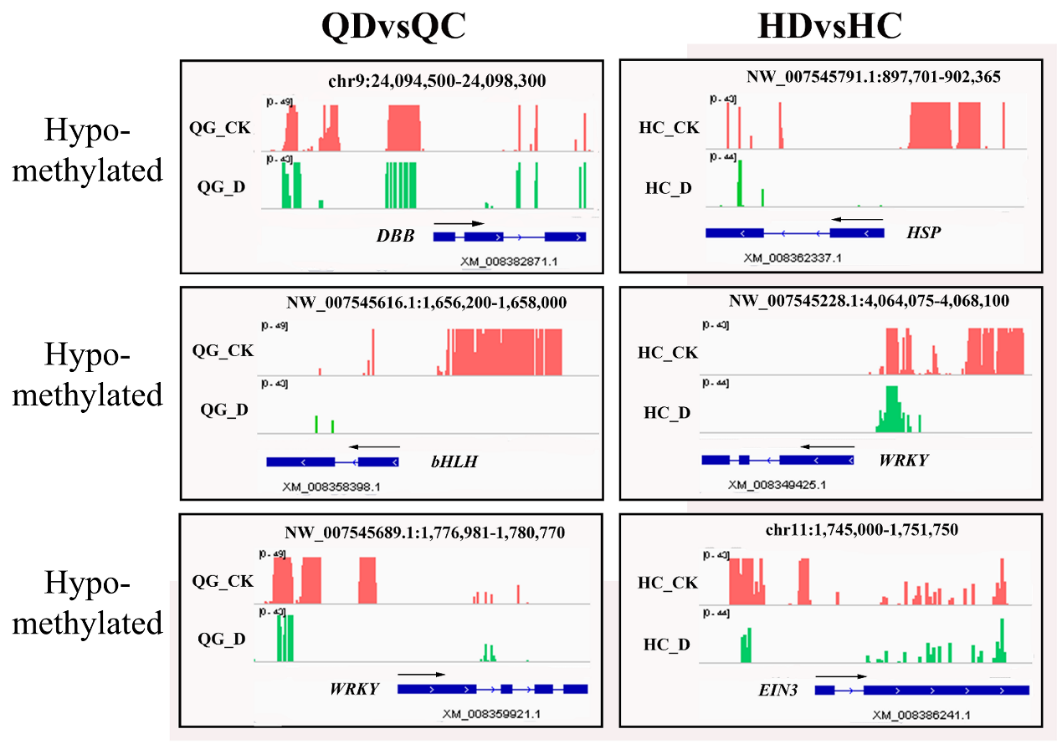


**Fig. S9** IGV snapshots of six differentially methylated transcriptional factors (TFs). QG_CK represents ‘Qinguan’ at control conditions; QG_D represents ‘Qinguan’ under water deficit treatment; HC_CK represents ‘Honeycrisp’ at control conditions, HC_D represents ‘Honeycrisp’ under water deficit stress.


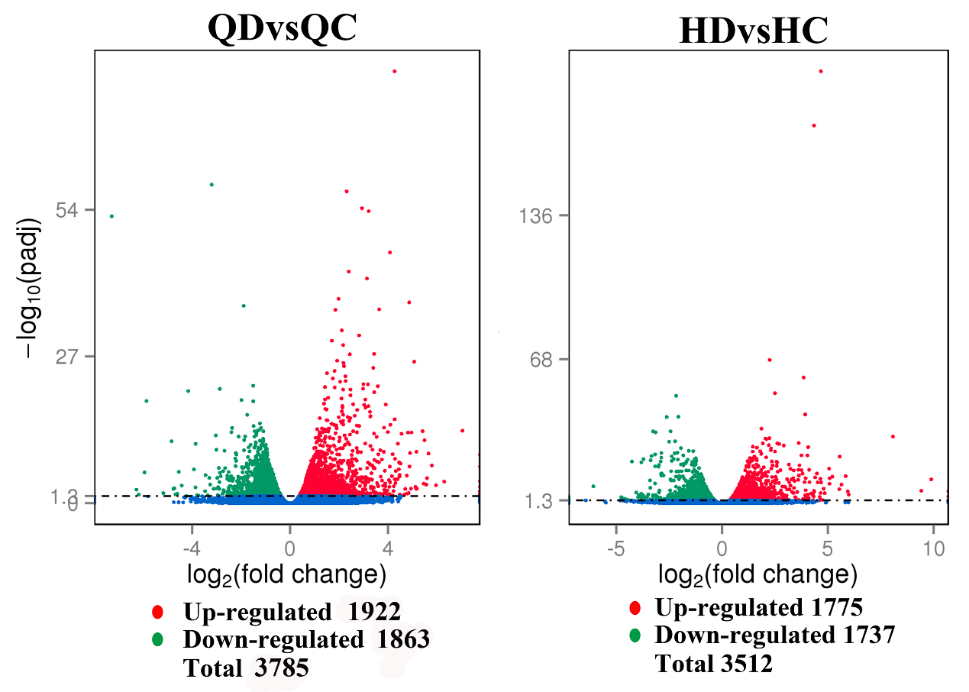


**Fig. S10** Differentially expressed genes (DEGs) in ‘Qinguan’ or ‘Honeycrisp’ in response to water deficit stress. Each dot represents one gene. The red dots represent up-regulated genes and the green dots represent down-regulated genes. The blue dots represent genes without differential expression. The X-axis is the log_2_ value of fold change and the Y-axis is the log_10_ value of the P-value. QC represents ‘Qinguan’ at control conditions; QD represents ‘Qinguan’ under water deficit treatment; HC represents ‘Honeycrisp’ at control conditions, HD represents ‘Honeycrisp’ under water deficit stress.
