# Supplementary material for: Single‐base methylome analysis reveals dynamic epigenomic differences associated with water deficit in apple
Source: Plant Biotechnol J. 2017 Sep 22;16(2):672–87. doi: 10.1111/pbi.12820 (PMC5787839; doi:10.1111/pbi.12820)
Supplement: Supplementary file 2 — Table S1 Homologs of DNA methyltransferase and demethylase proteins in apple. Table S2 Percentage of methylation levels of ‘Qinguan’ and ‘Honeycrisp’. Table S3 Methylation differences of dehydration‐related genes in ‘Qinguan’ and ‘Honeycrisp’ under water deficit. Table S4 Primers used for qRT‐PCR in this study. [file PBI-16-672-s002.docx]

**Table S1** Homologs of DNA methyltransferase and demethylase proteins in apple.

| Protein | Arabidopsis (GenBank) | Length (aa) | Malus domestica (GenBank) | Length (aa) | Chromosome |
| --- | --- | --- | --- | --- | --- |
| MET1 | AAA32829.1 | 1534 | AFV99138.1 | 1,570 | - |
|  |  |  | XP_008361333.1 | 1,574 | 15 |
| CMT1 | AEE36442.1 | 791 | null | - | - |
| CMT2 | AAK69757.1 | 1244 | [XP_017188227.1](https://www.ncbi.nlm.nih.gov/protein/1039882319?report=genbank&log$=prottop&blast_rank=4&RID=4ESV6C42014) | 860 | 6 |
|  |  |  | [XP_008346523.1](https://www.ncbi.nlm.nih.gov/protein/658022233?report=genbank&log$=prottop&blast_rank=5&RID=4ESV6C42014) | 611 | 6 |
| CMT3 | AAK69756.1 | 839 | [XP_017192106.1](https://www.ncbi.nlm.nih.gov/protein/1039841815?report=genbank&log$=prottop&blast_rank=1&RID=4ESV6C42014) | 855 | 13 |
|  |  |  | [NP_001280801.1](https://www.ncbi.nlm.nih.gov/protein/658309668?report=genbank&log$=prottop&blast_rank=2&RID=4ESV6C42014) | 1,096 | 13 |
|  |  |  | [XP_017192106.1](https://www.ncbi.nlm.nih.gov/protein/1039841815?report=genbank&log$=prottop&blast_rank=1&RID=4ETU9ARE015) | 855 | 13 |
|  |  |  | [AFV99137.1](https://www.ncbi.nlm.nih.gov/protein/411012961) | 974 | - |
| DRM1 | AED92154.1 | 624 | XP_017192312.1 | 741 | 13 |
| DRM2 | AED92056.1 | 626 | [XP_008379233.1](https://www.ncbi.nlm.nih.gov/protein/657974792?report=genbank&log$=prottop&blast_rank=1&RID=4EUJP3NR014) | 600 | 9 |
|  |  |  | [XP_017179298.1](https://www.ncbi.nlm.nih.gov/protein/1039861169?report=genbank&log$=prottop&blast_rank=3&RID=4EUJP3NR014) | 769 | 17 |
|  |  |  | [NP_001280787.1](https://www.ncbi.nlm.nih.gov/protein/658309551?report=genbank&log$=prottop&blast_rank=2&RID=4EUJP3NR014) | 930 | 17 |
|  |  |  | [XP_017182081.1](https://www.ncbi.nlm.nih.gov/protein/1039911030?report=genbank&log$=prottop&blast_rank=4&RID=4EUJP3NR014) | 505 | 13 |
| DDM1 | AAD28303.1 | 764 | [XP_008338101.1](https://www.ncbi.nlm.nih.gov/protein/658005900?report=genbank&log$=prottop&blast_rank=1&RID=4EUYUR1A015) | 740 | 15 |
|  |  |  | [XP_008338506.1](https://www.ncbi.nlm.nih.gov/protein/657948593?report=genbank&log$=prottop&blast_rank=2&RID=4EUYUR1A015) | 740 | 2 |
| DME | AED90760.1 | 1729 | [XP_008381296.1](https://www.ncbi.nlm.nih.gov/protein/657978707?report=genbank&log$=prottop&blast_rank=4&RID=4EVU0NFZ014) | 2,014 | 9 |
| ROS1 | AAP37178.1 | 1393 | [XP_008391745.1](https://www.ncbi.nlm.nih.gov/protein/657998670?report=genbank&log$=prottop&blast_rank=1&RID=4EVU0NFZ014) | 1,808 | 14 |
|  |  |  | [XP_008386935.1](https://www.ncbi.nlm.nih.gov/protein/657989466?report=genbank&log$=prottop&blast_rank=2&RID=4EVU0NFZ014) | 1,802 | 12 |
|  |  |  | [XP_008386936.1](https://www.ncbi.nlm.nih.gov/protein/657989466?report=genbank&log$=prottop&blast_rank=2&RID=4EVU0NFZ014) | 1,779 | 12 |
|  |  |  | [XP_008386937.1](https://www.ncbi.nlm.nih.gov/protein/657989466?report=genbank&log$=prottop&blast_rank=2&RID=4EVU0NFZ014) | 1,778 | 12 |

| **Table S2 Percentage of methylation levels of ‘Qinguan’ and ‘Honeycrisp’.** | | | | | | | | | | | | |
| --- | --- | --- | --- | --- | --- | --- | --- | --- | --- | --- | --- | --- |
| **Sample** | **C (Mb)** | **mC (Mb)** | **mC %** | **CG (Mb)** | **mCG (Mb)** | **mCG %** | **CHG (Mb)** | **mCHG (Mb)** | **mCHG %** | **CHH (Mb)** | **mCHH (Mb)** | **mCHH %** |
| **QG_CK1** | 3,121.50 | 557.3 | 17.86% | 378.9 | 203.2 | 53.62% | 413.9 | 155.8 | 37.65% | 2,328.60 | 198.3 | 8.52% |
| **QG_CK2** | 3,005.40 | 545.9 | 18.17% | 373.3 | 196.2 | 52.56% | 404.1 | 147.3 | 36.45% | 2,227.90 | 202.4 | 9.08% |
| **QG_D1** | 2,864.10 | 510.2 | 17.81% | 343.5 | 191.5 | 55.75% | 374.9 | 147.9 | 39.44% | 2,145.70 | 170.9 | 7.96% |
| **QG_D2** | 2,967.30 | 517.7 | 17.45% | 357.9 | 193.1 | 53.96% | 390.9 | 148.4 | 37.98% | 2,218.60 | 176.2 | 7.94% |
| **HC_CK1** | 3,091.70 | 514.7 | 16.65% | 373.8 | 185.4 | 49.60% | 407.3 | 136.7 | 33.56% | 2,310.70 | 192.7 | 8.34% |
| **HC_CK2** | 2,894.60 | 503.6 | 17.40% | 355.5 | 182 | 51.20% | 386.9 | 140.5 | 36.32% | 2,152.20 | 181.1 | 8.41% |
| **HC_D1** | 3,238.90 | 544.8 | 16.82% | 384.5 | 201.6 | 52.43% | 419.3 | 148.6 | 35.44% | 2,435 | 194.5 | 7.99% |
| **HC_D2** | 3,045.60 | 521.4 | 17.12% | 373.8 | 192.8 | 51.59% | 406.5 | 143.7 | 35.36% | 2,265.20 | 184.8 | 8.16% |

QG_CK represents Qinguan at control conditions; QG_D represents Qinguan under water deficit treatment;

HC_CK represents Honeycrisp at control conditions, HC_D represents Honeycrisp under water deficit stress.

1 and 2 in CK1, CK2, D1, D2 denote relevant replicates.

**Table S3 Methylation differences of dehydration-related genes in ‘Qinguan’ and ‘Honeycrisp’ under water deficit**

| Gene ID | Description | | DMR-Chromosome location (QDvsQC) | Trends-DMR region ( QDvsQC) | Methylation difference (QDvsQC) | DMR-Chromosome location (HDvsHC) | Trends-DMR region (HDvsHC) | Methylation difference (HDvsHC) |
| --- | --- | --- | --- | --- | --- | --- | --- | --- |
| Both in QDvsQC and HDvsHC | | | |  |  |  |  |  |
| 103411598 | dehydration-responsive protein RD22-like | | NW_007545254.1: 404615-404706 | Hyper-intron | 0.211 | NW_007545254.1: 414628-415052 | Hypo-intron | -0.143 |
| 103411598 | dehydration-responsive protein RD22-like | |  | = | - | NW_007545254.1: 405200-405389 | Hypo-intron | -0.114 |
| 103411598 | dehydration-responsive protein RD22-like | |  | = | - | NW_007545254.1: 403078-404686 | Hypo-exon,intron | -0.121 |
| 103411598 | dehydration-responsive protein RD22-like | |  | = | - | NW_007545254.1: 415223-415236 | Hyper-intron | 0.222 |
| 103413601 | dehydration-responsive element-binding protein 2F-like | | NW_007545326.1: 480867-481741 | Hyper-exon | 0.199 | NW_007545326.1: 481290-481877 | Hypo-exon | -0.176 |
| 103413601 | dehydration-responsive element-binding protein 2F-like | | NW_007545326.1: 483024-483068 | Hyper-promoter | 0.103 |  | = | - |
| 103413601 | dehydration-responsive element-binding protein 2F-like | | NW_007545326.1: 483165-483226 | Hypo-promoter | -0.304 |  | = | - |
| 103434826 | protein DEHYDRATION-INDUCED 19-like | | chr5: 4647207-4647440 | Hyper-promoter | 0.140 | chr5: 4647208- 4647523 | Hypo-promoter | -0.111 |
| 103437701 | protein EARLY RESPONSIVE TO DEHYDRATION 15-like | | chr1: 21365774- 21365850 | Hyper-promoter | 0.276 | chr1: 21365726- 21365877 | Hyper-promoter | 0.142 |
| 103443406 | dehydration-responsive protein RD22-like | | chr9: 14607725- 14607746 | Hyper-promoter | 0.535 | chr9: 14608860- 14608886 | Hyper-promoter | 0.125 |
| 103449730 | dehydration-responsive element-binding protein 2F | | chr12: 6849380- 6849512 | Hypo-promoter | -0.332 | chr12: 6849549- 6849933 | Hyper-promoter | 0.118 |
| 103455074 | protein DEHYDRATION-INDUCED 19 homolog 5-like | | chr14: 23114492- 23114705 | Hypo-promoter | -0.245 | chr14: 23114964- 23114979 | Hypo-promoter | -0.107 |
| 103455074 | protein DEHYDRATION-INDUCED 19 homolog 5-like | | chr14: 23110701- 23111461 | Hypo- exon,intron | -0.225 | chr14: 23114553- 23114778 | Hyper-promoter | 0.168 |
| 103455074 | protein DEHYDRATION-INDUCED 19 homolog 5-like | |  | = | - | chr14: 23114778- 23110347 | Hyper-intron | 0.146 |
| 103455074 | protein DEHYDRATION-INDUCED 19 homolog 5-like | |  | = | - | chr14: 23110675- 23111446 | Hyper- exon,intron | 0.218 |
| Only in QDvsQC | |  |  |  |  |  |  |  |
| 103410940 | dehydration-responsive protein RD22-like | | NW_007545243.1: 382499-382556 | Hyper-exon | 0.939 |  |  |  |
| 103420081 | protein EARLY RESPONSIVE TO DEHYDRATION 15-like, transcript variant X1 | | NW_007545583.1: 310715-310918 | Hyper-promoter | 0.104 |  |  |  |
| 103424730 | dehydration-responsive protein RD22-like | | NW_007545816.1: 4089663-4089740 | Hypo-promoter | -0.241 |  |  |  |
| 103428237 | dehydration-responsive element-binding protein 1E-like | | NW_007546008.1: 913925-914002 | Hypo-promoter | -0.114 |  |  |  |
| 103428237 | dehydration-responsive element-binding protein 1E-like | | NW_007546008.1: 912872-912933 | Hyper-promoter | 0.148 |  |  |  |
| 103428237 | dehydration-responsive element-binding protein 1E-like | | NW_007546008.1: 913119-913144 | Hypo-promoter | -0.110 |  |  |  |
| 103440344 | dehydration-responsive element-binding protein 2D-like | | chr8: 1450239- 1451316 | Hypo-promoter | -0.113 |  |  |  |
| 103453852 | dehydration-responsive element-binding protein 2B-like | | chr13:33940934- 33941129 | Hyper-promoter | 0.247 |  |  |  |
| Only in HDvsHC | | |  |  |  |  |  |  |
| 103400926 | dehydration-responsive protein RD22-like | |  |  |  | chr15: 15995919- 15996048 | Hyper-intron | 0.209 |
| 103400926 | dehydration-responsive protein RD22-like | |  |  |  | chr15: 15995584- 15995586 | Hyper-intron | 0.116 |
| 103410461 | dehydration-responsive element-binding protein 2D-like | |  |  |  | NW_007545217.1:1411403-1411665 | Hyper-promoter | 0.258 |
| 103422385 | protein DEHYDRATION-INDUCED 19-like, transcript variant X1 | |  |  |  | NW_007545714.1:1390856-1391169 | Hypo-intron | -0.119 |
| 103425005 | dehydration-responsive element-binding protein 1A-like | |  |  |  | NW_007545821.1: 437555-437567 | Hyper-promoter | 0.136 |
| 103439707 | dehydration-responsive element-binding protein 1D-like | |  |  |  | chr7: 23106567- 23106629 | Hypo-promoter | -0.138 |
| 103440205 | dehydration-responsive element-binding protein 1B-like | |  |  |  | chr1:25153166-25153235 | Hypo-promoter | -0.112 |
| 103441317 | dehydration-responsive protein RD22 | |  |  |  | chr8:15106888-15106976 | Hypo-intron | -0.170 |
| 103441317 | dehydration-responsive protein RD22 | |  |  |  | chr8:15106578-15106803 | Hyper-exon, intron | 0.198 |
| 103443449 | protein DEHYDRATION-INDUCED 19 homolog 5-like, transcript variant X2 | |  |  |  | chr9:15194307-15194703 | Hyper-intron | 0.127 |
| 103450572 | dehydration-responsive element-binding protein 2D-like | |  |  |  | chr12:21662210-21662667 | Hypo-promoter | -0.120 |

DMR means differentially methylated regions. QC represents Qinguan at control conditions, QD represents Qinguan under water deficit treatment; HC represents Honeycrisp at control conditions, HD represents Honeycrisp under water deficit stress.

**Table S4** Primers used for qRT-PCR in this study.

| *MdMET1*-qRT-F | TCCACTGGACAGATAGTTGACTTGA |
| --- | --- |
| *MdMET1*-qRT-R | GGCGCTTGGCTGTGTTG |
| *MdDRM1*-qRT-F | AGCCCCCAGACGCCATA |
| *MdDRM1*-qRT-R | AGGCCACCATTTCCTTGTCA |
| *MdDRM2*-qRT-F | GGAGCCCGTGCAACAATCTA |
| *MdDRM2*-qRT-R | CCTCCAGTCCATCACGATGA |
| *MdDRM3*-qRT-F | CGAGAATAGGTTTCGCATTGTTC |
| *MdDRM3*-qRT-R | TGTGGCATTGCATCCTCAA |
| *MdCMT2*-qRT-F | GGAGCAAATTTCCGGGATCTAC |
| *MdCMT2*-qRT-R | CTCTCTCCGAGCCACATTGTC |
| *MdCMT3*-qRT-F | AATATTCGCTTGCGGGTTTG |
| *MdCMT3*-qRT-R | CAGACACGGTGGCCATCAC |
| *MdCMT3c*-qRT-F | GGTGCCTATGGGCTTCCA |
| *MdCMT3c*-qRT-R | CGGGCCCCCCACAA |
| *MdDDM*-qRT-F | GCGGTATGAAAGGAAAGCTTAACA |
| *MdDDM*-qRT-R | GGTCAGGATGATTGCAGTTCTTC |
| *MdROS*-qRT-F | CCACCTGACCAAGCAAAAGAGTA |
| *MdROS*-qRT-R | CACGCTTTTCAACCCCAATC |
| *MdDME*-qRT-F | CAAGTTAATGAGATGTTCGCAGATC |
| *MdDME*-qRT-R | CACCCCCTTGGAACATCAAT |
| *MdEF*-qRT-F | ATTCAAGTATGCCTGGGTGC |
| *MdEF*-qRT-R | CAGTCAGCCTGTGATGTTCC |
